# A pendulum of induction between the epiblast and extra-embryonic endoderm supports post-implantation progression

**DOI:** 10.1242/dev.192310

**Published:** 2022-08-22

**Authors:** Erik J. Vrij, Yvonne S. Scholte op Reimer, Laury Roa Fuentes, Isabel Misteli Guerreiro, Viktoria Holzmann, Javier Frias Aldeguer, Giovanni Sestini, Bon-Kyoung Koo, Jop Kind, Clemens A. van Blitterswijk, Nicolas C. Rivron

**Affiliations:** 1 MERLN Institute for Technology-inspired Regenerative Medicine, Maastricht University, Universiteitssingel 40, 6229 ER Maastricht, Netherlands; 2 Institute of Molecular Biotechnology of the Austrian Academy of Sciences, Vienna Biocenter, Dr. Bohr-Gasse 3, 1030 Vienna, Austria; 3 Hubrecht Institute, Royal Netherlands Academy of Arts and Sciences (KNAW) and University Medical Center Utrecht, UtrechtUppsalalaan 8, 3584 CT Utrecht, Netherlands; 4 Department of Molecular Biology, Faculty of Science, Radboud Institute for Molecular Life Sciences, Radboud University Nijmegen, Geert Grooteplein Zuid 10, 6525 GA Nijmegen, Netherlands

**Keywords:** Blastoids, Primitive endoderm, Extra-embryonic endoderm/epiblast rosette, Post-implantation development, Embryonic stem cells, Pro-amniotic cavity

## Abstract

Embryogenesis is supported by dynamic loops of cellular interactions. Here, we create a partial mouse embryo model to elucidate the principles of epiblast (Epi) and extra-embryonic endoderm co-development (XEn). We trigger naive mouse embryonic stem cells to form a blastocyst-stage niche of Epi-like cells and XEn-like cells (3D, hydrogel free and serum free). Once established, these two lineages autonomously progress in minimal medium to form an inner pro-amniotic-like cavity surrounded by polarized Epi-like cells covered with visceral endoderm (VE)-like cells. The progression occurs through reciprocal inductions by which the Epi supports the primitive endoderm (PrE) to produce a basal lamina that subsequently regulates Epi polarization and/or cavitation, which, in return, channels the transcriptomic progression to VE. This VE then contributes to Epi bifurcation into anterior- and posterior-like states. Similarly, boosting the formation of PrE-like cells within blastoids supports developmental progression. We argue that self-organization can arise from lineage bifurcation followed by a pendulum of induction that propagates over time.

## Abstract (precis)

**Summary:** A set of molecules directs the co-formation of blastocyst-stage primitive endoderm-like and epiblast-like cells in embryoid bodies and blastoids. This embryonic–extra-embryonic niche self-organizes and progresses through post-implantation-like stages via a sequence of mutual inductions.

## INTRODUCTION

In certain species, extrinsic positional cues create a pre-pattern for development, e.g. through the local deposition of maternal RNA on one side of a *Drosophila* egg. However, mammalian development appears to rather favor decentralized and regulative principles, termed self-organizing, that prevail over more deterministic behaviors (e.g. pre-patterned hard-wired genetic programs). Accordingly, 16-cell stage mouse blastomeres can be dissociated and re-aggregated to form a competent blastocyst in minimal medium (Tarkowski et al., 2010; Suwińska et al., 2008; Posfai et al., 2017). Such a logic leverages the properties of gene regulatory networks and molecular noise to achieve cellular decision making (Semrau et al., 2017; Balázsi et al., 2011), and of non-linear cellular interactions to ensure lineage divergence and progression. In the mouse blastocyst, such principles are at play between the embryonic and extra-embryonic tissues (Arnold and Robertson, 2009), ensuring trophoblast/inner cell mass (Niwa et al., 2005) and epiblast (Epi)/primitive endoderm (PrE) development (Frankenberg et al., 2011; Bessonnard et al., 2014; Chazaud et al., 2006). This logic also supports organogenesis (Briscoe, 2019; Olson, 2006; Zuniga, 2015). To better understand these loops of cellular interactions, we created a partial mouse embryo model undergoing phenomenological self-organization and observed sequences of reciprocal inductions supporting its autonomous progression over time.

The early mammalian conceptus consists of three lineages: the pluripotent epiblast (Epi), which forms the embryo proper; and the two extra-embryonic lineages – the trophoblast and primitive endoderm (PrE) – that contribute to the placenta and yolk sac, respectively (Rossant and Tam, 2009; Lokken and Ralston, 2016). In mice, the bifurcation between PrE and Epi cells is established between E3.25 and E4.5 (Schrode et al., 2014; Onishi and Zandstra, 2015; Chazaud et al., 2006; Bassalert et al., 2018; Plusa et al., 2008), and is marked by the timed expression of the transcription factors Oct4, Nanog, Klf4 and Sox2 in the Epi (Neagu et al., 2020), and Gata6, Pdgfrα, Gata4, Sox17 and Sox7 in the PrE (Lokken and Ralston, 2016; Artus et al., 2013; Lo Nigro et al., 2017). Experiments suggest that PrE specification is initiated by lineage priming (Ohnishi et al., 2014) that exploits polycomb (Illingworth et al., 2016), chromatin modifier (Goolam and Zernicka-Goetz, 2017) and small-RNA (Ngondo et al., 2018) activities, along with the progression of gene regulatory networks (Lokken and Ralston, 2016) and intercellular signaling circuitries [e.g. FGF/Mapk/Erk (Azami et al., 2017; Kang et al., 2017; Molotkov et al., 2017; Ohnishi et al., 2014; Krawchuk et al., 2013; Schröter et al., 2015; Wigger et al., 2017; Chazaud et al., 2006; Yamanaka et al., 2010; Wicklow et al., 2014), Lif/Stat (Morgani and Brickman, 2015; Onishi and Zandstra, 2015), Nodal/Smad2/3 (Mesnard et al., 2006; Papanayotou and Collignon, 2014), Bmp4/Smad4 (Graham et al., 2014; Wang et al., 2004) and Wnt/β-catenin (Corujo-Simon et al., 2017; ten Berge et al., 2011) pathways]. The initial PrE cell specification is reinforced by Epi inductions made through FGF4 signaling (Mulvey et al., 2015; De Caluwé et al., 2019; Molotkov and Soriano, 2018; Artus et al., 2013; Frum and Ralston, 2015; Houston, 2017) to progressively lock cell fates, to promote their physical segregation, and to promote the epithelialization and lining of the PrE along the blastocoel cavity (Meilhac et al., 2009; Burtscher and Lickert, 2009; Saiz et al., 2013; Brimson, 2016). This process is regulative as it senses and adjusts the mutually allocated cell numbers (Plusa and Hadjantonakis, 2018; Grabarek et al., 2012; Mathew et al., 2019; Yamanaka et al., 2010). Here, we further explore the extent by which the Epi and PrE co-develop.

The use of microsystems to control cell numbers (Vrij et al., 2016a) and of chemically defined medium (Kubaczka et al., 2014) opens possibilities to increase the control, throughput and screening capacities of embryo models (Vrij et al., 2016a; Rivron et al., 2018a). Previously, we induced the formation of blastocyst-like structures by combining trophoblast stem cells (TSCs) and ESCs, which we termed blastoids (Rivron et al., 2018a). Blastoids generate PrE-like cells from the ESCs, as confirmed in later studies (Sozen et al., 2019; Posfai et al., 2021), and thus make up the three founding cell lineages. However, the limited expansion of the PrE-like cells is likely to restrict their potential to develop. Here, we run combinatorial screens of proteins, GPCR ligands and small molecules in a microwell array platform and in chemically defined conditions. This directs ESCs to rapidly and efficiently co-form blastocyst-stage PrE- and Epi-like cells. These cells then develop synergistically in minimal medium to form a structure resembling the post-implantation Epi and extra-embryonic endoderm tissues (XEn), referred to as Epi/XEn. We apply this model to test the share of autonomous development of the Epi/XEn module. We observe mutual inductions between the Epi and PrE that support the potential for growth, viability, specification and morphogenesis that underlie aspects of post-implantation development. We propose that development can be driven by sequences of reciprocal interactions between progressively diverging cell types.

## RESULTS

### Naive pluripotency enhances the ESCs potential for PrE differentiation

**Fig. 1.**
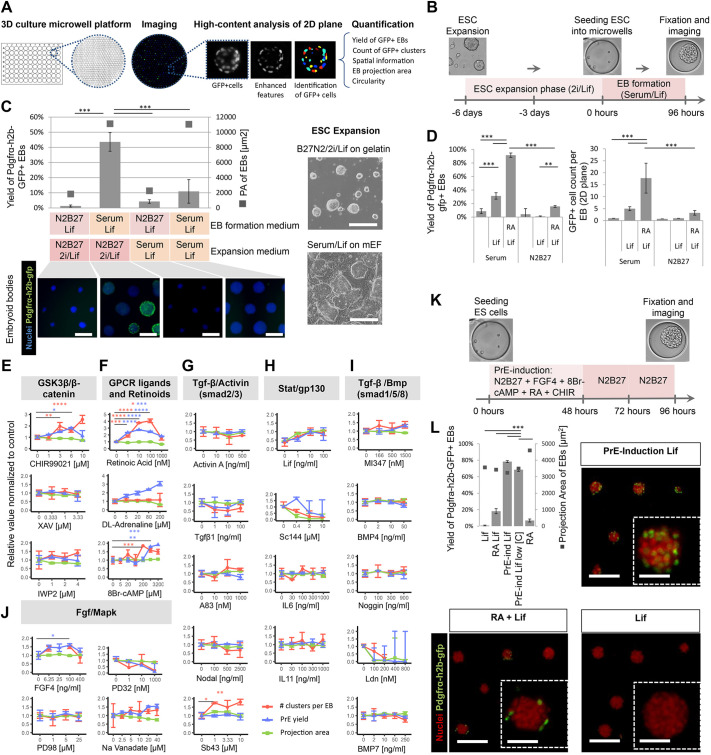
**The initial naive state of ESCs and specific signaling pathways induce efficient co-development of the PrE-/Epi-like niche *in vitro*.** (A) High-content screening (HCS) method for 96-well plates imprinted with agarose microwell arrays (430 microwells per well) in which EBs are formed (each microwell captures a single EB), cultured and imaged (2D mid-focal plane). (B) Schematic of experimental set-up, including ESC expansion and EB-based primitive endoderm (PrE) differentiation. (C) Top and right: quantified yield of PrE-differentiation (Pdgfrα^+^, left axis) and proxy for EB size (2D projection area, right axis) derived from ESCs expanded in naive (N2B27/2i/Lif) versus serum/Lif conditions. Bottom: fluorescence images show the nuclei (blue) and Pdgfrα-h2b-gfp^+^ clusters (green) within EBs formed by combinations of different formation and ESC expansion media. Bright-field images of ESCs expanded in N2B27/2i/Lif or serum/Lif [on mouse embryonic fibroblasts (mEFs)]. Scale bars: 200 µm. (D) Yield of Pdgfrα-h2b-gfp^+^ EBs and the number of GFP^+^ clusters per EB in N2B27 or serum media supplemented with or without Lif and with or without RA. Data are mean±s.d. obtained from *n*=4 wells, with each well containing ∼400 EBs. ANOVA with Bonferroni post-hoc test (***P<0.001, **P<0.01). (E-J) Dose-response curves showing the effect of different soluble pathway modulators after 96 h in culture on the yield of Pdgfrα-h2b-gfp^+^ EBs (blue), the number of Pdgfrα-h2b-gfp^+^ clusters per EB (red) in median focus plane (10× objective) and the EB projection area (as a proxy for EB size, green). All values were normalized to H_2_O/DMSO controls. Mean and s.d. values were obtained from *n*=3 or 4 wells, with every well containing ∼400 EBs. ANOVA with Tukey's multiple comparison test (****P<0.0001, ***P<0.001, **P<0.01, *P<0.05). (K) Schematic for chemically induced differentiation of EBs towards PrE. (L) Graph shows yields for PrE differentiation (left axis) and EB projection area (right axis) using the induction cocktail. Low [C] indicates lower concentrations of cAMP (1 mM) and CHIR99021 (3 µM). Representative fluorescent images of indicated conditions. PrE inductions in Lif and RA/Lif media are shown for comparison. Scale bars: 200 µm; 40 µm (insets). Data are mean±s.d. obtained from *n*=4 wells, with each well containing ∼400 EBs. ANOVA with Tukey's multiple comparison test (***P<0.001). Images in C, K and L are taken after 96 h of culture.

Forming tissues of appropriate size is crucial to ensure relevant concentrations and distributions of biological parameters (e.g. molecules and mechanical forces). We used a high-content screening platform of non-adherent hydrogel microwells in 96-well plates (Vrij et al., 2016b) to reproducibly aggregate small and defined numbers of ESCs that reflected the number of inner cells within blastocysts (Fig. 1A). The cell number followed a Poisson distribution across the 430 microwells (7-12 cells per microwell) and the cells aggregated within 24 h (Fig. 1B, Fig. S1). We quantified PrE differentiation via *in situ* imaging of a fluorescent reporter under the promoter for Pdgfrα (ESCs^Pdgfrα-h2b-gfp/+^, Fig. 1A) (Artus et al., 2010; Plusa et al., 2008). EBs survived in serum-free N2B27 medium supplemented with leukemia inhibitory factor (Lif) but did not proliferate and formed only a few Pdgfrα^+^ cells (yield of Pdgfrα^+^ EBs: 1%, Fig. 1C, Fig. S2). In contrast, the addition of serum induced the appearance and proliferation of Pdgfrα^+^ cells (44%, Fig. 1C, *P*<0.001). Consistent with a previous report (Schröter et al., 2015), we observed that an initial 2D expansion in chemically defined N2B27/2i/Lif medium supporting a naive pre-implantation-like state (Ying et al., 2008) enhanced the susceptibility for formation of Pdgfrα^+^ cells, when compared with expansion in serum-containing medium that captures concomitant peri-implantation-like populations (Neagu et al., 2020) (Fig. 1C). We concluded that, similar to the blastocyst cells (Artus et al., 2010; Plusa et al., 2008), formation of Pdgfrα^+^ cells is favored by an initial permissive state, along with signals present in serum that regulate specification and proliferation.

### A three-dimensional screen reveals signaling pathways that regulate Pdgfrα expression

Signaling molecules have been proposed to influence PrE specification, including Lif (Morgani and Brickman, 2015), retinoic acid (Cho et al., 2012), FGF (Yamanaka et al., 2010; Chazaud et al., 2006; Goldin and Papaioannou, 2003), GSK3β/β-catenin (Krawetz and Kelly, 2008; Price et al., 2013) and Nodal (Niakan et al., 2013; Mesnard et al., 2006). In the conceptus, these molecules activate pathways that are likely to act synergistically but investigating their respective interactions and functions remains difficult. We thus modulated these pathways in EBs. Although Lif (10 ng/ml) increased the yield of Pdgfrα^+^ EBs in serum cultures (30% yield, 3.6-fold increase, Fig. 1D), addition of retinoic acid (RA; 10 nM) further improved the process (91% yield, 3-fold increase, Fig. 1D) and increased the number of Pdgfrα^+^ clusters per EB (5.5-fold increase, Fig. 1D, clusters are defined as Pdgfrα^+^ cells found within the equatorial plane of EBs, see Materials and Methods). In contrast, the effect of these two molecules appeared restricted in serum-free N2B27/Lif medium (16% yield). Consistent with a synergistic action of multiple pathways, we concluded that Lif and RA support but are not sufficient to efficiently form Pdgfrα^+^ cells.

We then created a small library of activators and inhibitors of signaling pathways that are active in the blastocyst (Table S1). We first tested them individually in a serum-containing medium and measured the percentage of Pdgfrα^+^ EBs (yield) and the number of Pdgfrα^+^ clusters per EB. FGF4 (100 ng/ml) and the GSK3β inhibitor CHIR99021 (6 µM), which act on pathways active in the blastocyst Epi (ten Berge et al., 2011; Azami et al., 2019), increased the yield (44% and 81%, respectively) and the number of clusters per EB (both 1.6-fold; Fig. 1E,J). Inhibiting Wnt secretion (IWP2) and Wnt processing (XAV939) did not significantly affect specification (Fig. 1E). We concluded that the FGF and GSK3β/β-catenin pathways regulate Pdgfrα^+^ cell specification.

In contrast, although BMP signaling has been proposed to contribute to PrE development (Graham et al., 2014), activation of the SMAD pathway by activin A and Tgfβ1 elicited a decline, albeit non-statistically significant, of either the yield or Pdgfrα^+^ cell number. Consistently, the Tgfβ receptor inhibitor SB431542 and the Alk1/2 inhibitor ML347 (BMP signaling) enhanced the formation of Pdgfrα^+^ cells (Fig. 1I), whereas the BMP pathway inhibitor LDN193189 prevented proliferation (Fig. 1I). We concluded that ESCs might have lost the potential to respond to the Tgfβ signaling pathway or that this pathway acts on elements other than PDGFRa, thereby preventing detection of its effect. We concluded that the activation of the FGF and inhibition of the GSK3β and Tgfβ pathway facilitate the generation of Pdgfrα^+^ cells from naive ESCs.

### A three-dimensional screen reveals GPCR ligands inducing Pdgfrα expression

Next, to complement the action of classical developmental pathways, we investigated the potency of signaling through G-protein-coupled receptors (GPCR) by screening for 264 GPCR ligands, informed by previous findings that cAMP modulates Pdgfrα expression in EBs (Vrij et al., 2016a). DL-adrenaline, a β-adrenoceptor agonist acting upstream of the cAMP/PKA pathway, strongly increased the yield of Pdgfrα^+^ EBs (206%) without affecting the overall size of EBs or the number of clusters (Fig. 1E). Accordingly, 8Br-cAMP (3200 µM) also increased the yield of Pdgfrα^+^ EBs by 91% when compared with serum/Lif alone, Fig. 1E) (Vrij et al., 2016a) without affecting EBs size. We concluded that DL-adrenaline and cAMP potentiate naive ESCs for Pdgfrα expression independent of proliferation. Altogether, we concluded that FGF4, GSK3β/β-catenin, Lif, RA, DL-adrenaline and cAMP individually increase the expression of Pdgfrα.

### A combinatorial screen delineates a chemically defined medium inducing Pdgfrα expression

Because signaling molecules often act in concert, we ran combinatorials of molecules, this time in serum-free medium (N2B27 medium, Fig. S3A,B). Using a factorial design screening approach (Hutchens et al., 2007), we tested combinations of 8Br-cAMP, DL-Adrenaline, Lif, FGF4, sodium orthovanadate, CHIR99021, ML347, SB431542, RA and activin A at effective concentration ranges. Specific combinations preserved EB viability and integrity, and induced PrE-like specification (measured by EB projection area, circularity, and Pdgfrα and Gata6 expression, respectively; Figs S3C and S4). Among the selected 21 combinations, a medium containing 8Br-cAMP (1 mM), RA (10 nM), FGF4 (100 ng/ml) and CHIR99021 (5 µM) led to a stark upregulation of the yield of Pdgfrα^+^ EBs (78%, Fig. 1K,L, Fig. S4C). Consistent with the important role of RA (Niakan et al., 2013; Cho et al., 2012), depleting this molecule from the induction medium reduced the yield significantly (Fig. 1L). However, the synergy with other factors was essential for robust and efficient induction (Fig. 1L). This chemically defined inductive medium also reduced the number of dead cells per EB to levels similar to serum-containing medium (Fig. S5B), and cells no longer required the presence of Lif for maintaining viability or Pdgfrα expression (Fig. S5A).

### Formation of a partial blastocyst model with PrE- and Epi-like cells

**Fig. 2.**
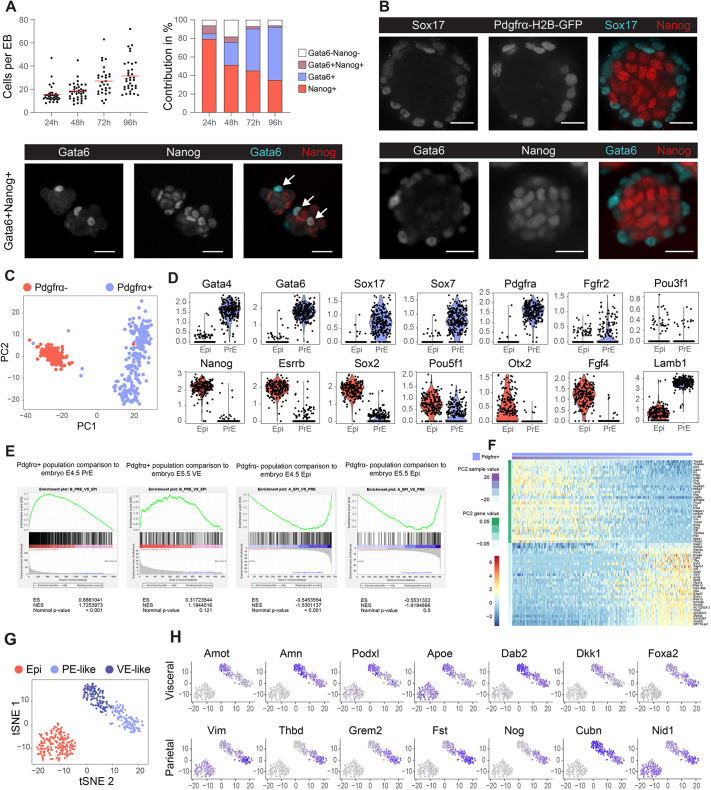
**EBs form a niche that includes both Epi- and PrE-like cells with putative PE and VE populations.** (A) Total cell numbers per PrE-induced EB at 24, 48, 72 and 96 h (left), and associated average contribution of double-positive (Nanog^+^, Gata6^+^), double-negative (Gata6^−^, Nanog^−^), Gata6^+^ and Nanog^+^ cells per EB over time (right). The image depicts double-positive (Gata6^+^ and Nanog^+^, white arrows) cells found within PrE-induced EBs at 24 h (confocal spinning disk fluorescence image, single plane). EBs were randomly selected and pooled from *n*=3 wells. (B) Immunofluorescence images of Sox17, Pdgfrα-H2B-GFP, Nanog and Gata6 of PrE-induced EBs after 96 h of culture. Scale bars: 50 µm. (C) Principal component analysis of single-cell transcriptomics data for PrE-induced EBs after 96 h of culture in microwells. (D) Violin plots of RNA normalized transcript counts for PrE and Epi markers found in Pdgfrα^+^ cells (PrE) and Pdgfrα^−^ cells (Epi). (E) Gene set enrichment analysis (GSEA) comparing the gene expression signature of the Pdgfrα^+^ (first and second images) and Pdgfrα^−^ (third and fourth images) cell cluster to mouse embryo E4.5 PrE, E5.5 VE, E4.5 Epi and E5.5 Epi (Mohammed et al., 2017). ES, enrichment score; NES, normalized enrichment score. (F) Heatmap depicting single-cell RNA expression data of the top and bottom 30 most differentially expressed genes along the PC2 axis in the subpopulation of Pdgfrα^+^ cells. (G) tSNE mapping delineates three putative subpopulations: E4.5 Epi, early VE and early PE. (H) tSNE maps for the early VE genes *Amot*, *Amn*, *Podxl*, *Apoe*, *Dab2*, *Dkk1* and *Foxa2*, and for the PE genes *Vim*, *Thbd*, *Grem2*, *Fst*, *Nog*, *Cubn* and *Nid1*. Axes labels are tSNE dimension 1 (vertical) and 2 (horizontal). Color intensity correlates with expression level.

Within 24 h of induction, double-positive (Nanog^+^/Gata6^+^) cells and double-negative cells emerged in a salt and pepper-like distribution between Nanog^+^/Gata6^−^ cells, as observed in the E3.5 blastocyst (Chazaud et al., 2006; Saiz et al., 2020) (Fig. 2A, Fig. S6). Over time, the relative number of Gata6^+^ cells increased (Fig. 2A, Figs S6 and S7) and the initially intermingled cell types spontaneously segregated to form an outer layer of cells expressing Gata6 (Meng et al., 2018; Schrode et al., 2014; Wang et al., 2010; Cai et al., 2008; Lavial et al., 2012) and Sox17 (Qu et al., 2008; Kinoshita et al., 2015; Artus et al., 2011), and inner Nanog^+^ cells (96 h, Fig. 2B and Fig. S8), consistent with the segregation of the PrE and Epi that occurs in the E4.5 blastocyst (Chazaud et al., 2006). The observation of robust proportioning of Nanog^+^ and Gata6^+^ cells, despite exposure to inductive molecules, might point at regulatory circuits ensuring a balance between the two cell types, as previously proposed (Raina et al., 2020), and was disturbed upon FGF/Mapk/Erk signaling inhibition (Fig. S9). Notably, the chemically defined inductive medium and its individual components did not interfere with Epi and PrE cell specification in mouse blastocysts (Fig. S10), thereby suggesting that additional layers of regulation prevent unbalancing of these cell numbers.

We then characterized the Epi- and PrE-like cells by isolating them based on Pdgfrα antibody labeling and analyzing them via single-cell transcriptomics (96 h). Principal component (PC) analysis showed two distinct subpopulations along the PC1 axis that corresponded to the Pdgfrα^−^ and Pdgfrα^+^ cells (Fig. 2C), with the top differentially expressed genes reminiscent of those for Epi- and PrE-like cells, respectively (Fig. S11A). The Pdgfrα^+^ cells expressed *Gata6*, *Gata4*, *Pdgfra*, *Sox7*, *Fgfr2* and *Sox17* at higher levels than the Pdgfrα^−^ cells, which preferentially expressed E4.5 Epi genes such as *Nanog*, *Sox2*, *Esrrb*, *Fgf4* and *Oct4* (Fig. 2D). Gene set enrichment analysis (GSEA) comparing the Pdgfrα^+^ and Pdgfrα^−^ cells with PrE cells from E4.5 mouse embryos (Mohammed et al., 2017) showed statistically significant (*P*<0.001) enrichment scores of 0.686 and −0.545, respectively (Fig. 2E). In contrast, these cells were not significantly enriched in the transcripts of peri-implantation stage VE cells (E5.5, enrichment score of 0.317, gene list in Table S3). We concluded that these two cellular populations best reflect the Epi and PrE at a peri-implantation blastocyst stage.

However, we observed that, in contrast to the relatively homogeneous transcriptome of the Epi-like cells, the PrE-like cells were scattered along the PC2 axis (Fig. S11A,B, Table S2). Additional analysis showed that, although they reflected E4.5 PrE rather than E5.5 visceral endoderm (VE) cells, they were primed for the peri-implantation divergence occurring around E5.0 between the parietal endoderm (PE)-expressing markers [such as *Fst* (follistatin) and *Vim* (vimentin)] and VE-expressing the markers [*Dab2* and *Podxl* (podocalyxin)] (Fig. 2F). tSNE clustering also revealed these two PrE subpopulations (Fig. 2G, Fig. S12A) with mutually distinct expression levels of PE genes (Edgar et al., 2013), such as *Vim*, *Fst*, *Thbd*, *Sema6* and *Nog*, and VE genes (Edgar et al., 2013; Pfister et al., 2007) such as *Amn*, *Cubn*, *Dab2*, *Podxl* and *Apoe* (Fig. 2H, Fig. S12B). Compared with the VE-like subpopulation, the PE-like subpopulation showed higher expression levels for extracellular matrix (ECM) proteins, including *Col4a1*, *Col4a2*, *Nid1*, *Lama1*, *Lamc1* and *Sparc* (Fig. S12A), which are necessary for the deposition of a thick multilayered basal lamina, named Reichert's membrane, along the inner side of the trophoblasts (Salamat et al., 1995). We produced a list of differentially expressed genes that may be used as potential early markers for PE and VE (Figs S11B and S12A, Table S2). From these data, we performed gene ontology term analysis (Table S2). In the VE-like subpopulation, genes encoding cell polarity regulators that are typical of an epithelium (e.g. *Jam3*, *Cfl1*, *Lmna*, *Amot* and *Gja1*) and of a response to Tgfβ pathway activation (*Dab2* and *Runx1*) were enriched when compared with those in the Epi. We concluded that, beyond an intrinsic program regulated by Gata6 (Morrisey et al., 1998; Cai et al., 2008), the Epi might induce Tgfβ activity in the VE, a pathway that often regulates epithelialization. Of note, a role for Nodal has previously been proposed later on (at E5.0) during the peri-implantation stage for VE specification (Mesnard et al., 2006; Edgar et al., 2013; Pfister et al., 2007). Altogether, this model points to the neutrality of Nodal, activin, Tgfb1, BMP4 or BMP7 in the initial specification of the PrE but to a possible role for Tgfβ pathways in the initiation of the VE.

Overall, we concluded that the chemically defined medium induced co-formation and spatial organization of blastocyst-stage PrE- and Epi-like cells, the former being primed for bifurcating into both VE and PE lineages. These populations recapitulate known intercellular signaling circuitries, including Epi-produced FGF4 that contributes to PrE specification and Tgfβ superfamily members that shape the VE.

### The PrE/Epi model progresses into a post-implantation rosette and pro-amniotic-like cavity in minimal conditions

**Fig. 3.**
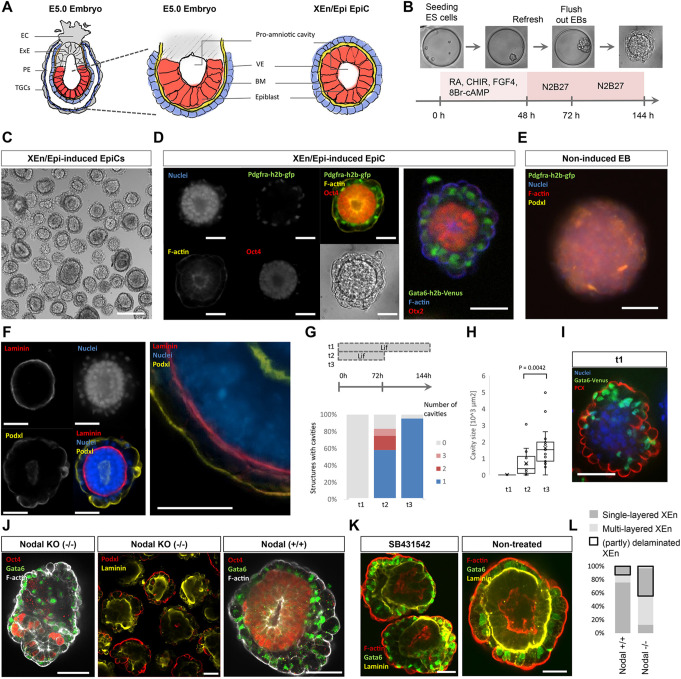
**The PrE-/Epi-like niche spontaneously progresses into a post-implantation extra-embryonic endoderm/epiblast epithelialized pro-amniotic-like cavity (XEn/Epi EpiC) in minimal culture conditions.** (A) Schematic depicting an E5.0 conceptus (left, middle) and corresponding tissues in an XEn/Epi EpiC (right). EC, ectoplacental cone; ExE, extra-embryonic ectoderm; PE, parietal endoderm; TGCs, trophoblast giant cells; VE, visceral endoderm; BM, basal lamina. (B) Schematic for XEn/Epi EpiC formation. (C) Bright-field image of XEn/Epi rosettes and EpiCs. Scale bar: 200 µm. (D) Immunofluorescence and bright-field images of individual XEn/Epi rosette images after 120 h of culture. Staining for nuclei (DNA), F-actin (pro-amniotic cavity), Pdgfrα-h2b-gfp (PrE), Oct4 (pluripotent Epi) (left) and Otx2 (right). (E) EB cultured under the same basic conditions but without PrE-induction molecules. (F) Immunofluorescence images depicting cell nuclei (DNA), Podxl (polarization) and laminin (basal lamina) in a XEn/Epi pro-amniotic-like cavity. (G,H) Effect of Lif on (G) the percentage of structures forming a pro-amniotic cavity or multiple cavities and (H) the resulting integrated surface area of the cavities. *P*-value calculated according to the Mann–Whitney *U*-test. Boxes and whiskers indicate the first, median and third quartile, and minimum and maximum data points excluding outliers, respectively. This result was repeatedly replicated (>10 times) in other experiments as inclusion of a negative control. (I) Immunofluorescence image of a non-cavitated and non-polarized structure resulting from continuous Lif supplementation, labeled for nuclei, Gata6 (PrE) and Podxl (polarization). (J) Immunofluorescence images of 120 h XEn/Epi EpiCs from double Nodal knockout (−/−) and control (+/+) ESCs. (K) Immunofluorescence images of 120 h XEn/Epi EpiCs treated with the Nodal/activin signaling inhibitor SB431542 and non-treated controls. (L) Percentage of structures (32 in total) that contained a laminated or delaminated XEn layer (outlined in black) that is either single or multilayered. Scale bars in D-F,I-K: 50 µm.

*In utero*, Epi rosettes form at the time of blastocyst implantation. This coincides with the deposition of a laminin-rich basal lamina by the PrE that polarizes the underlying Epi and triggers the formation of the pro-amniotic cavity (Fig. 3A) (Li et al., 2003). Accordingly, Epi-like rosettes can form in the absence of PrE cells when ESCs are encapsulated in Matrigel and cultured in serum-containing medium (Moore et al., 2014; Bedzhov and Zernicka-Goetz, 2014). To assess the potential of the blastocyst PrE/Epi model to autonomously progress, we washed it and cultured it in minimal N2B27 medium. The cells proliferated and underwent morphogenesis by forming a rosette that progressed into a cavity morphologically resembling the polarized Epi/XEn tissue (Fig. 3A-C). We termed these structures EpiCs (Epi/XEn pro-amniotic-like cavities). On the contrary, the Epi/PrE-like model maintained in the initial specification culture medium did not efficiently undergo morphogenesis. In addition, aggregates of ESCs alone did not proliferate in such minimal medium (data not shown). Similar to post-implantation embryos, the rosette-like cells expressed Oct4 and Otx2, and accumulated F-actin and Podxl at the apical side (Fig. 3D,E), while the PrE-like cells produced a laminin-rich basal lamina and also became polarized (Podxl, Fig. 3E). Over time, the cavities increased in size (Fig. S13A). The process was both efficient (94%) and reproducible (Fig. 3F, Fig. S13B). We concluded that the two tissues mutually supported their proliferation and morphogenesis, and that a switch of signaling environment is necessary for post-implantation transition.

### Lif signaling inhibits the formation of the pro-amniotic-like cavity

Because progression required a switch of signaling environment, we then tested factors that might act as developmental checkpoints. Lif has been shown to prevent the formation of the cavity in Matrigel-embedded/serum-cultured conditions (Shahbazi et al., 2017). Similarly, we observed that the presence of Lif during the first 3 days or for the entire 6 days of *in vitro* development reduced and abrogated, respectively, the formation of the pro-amniotic cavity, as seen by the absence of Podxl within the Epi-like cells and the arched bilateral/apical location of Podxl in the VE-like cells (Fig. 3F-H). In addition, as previously observed (Moore et al., 2014; Bedzhov and Zernicka-Goetz, 2014), the inhibition of apoptosis using Z-vad-fmk did not impair lumenogenesis (Fig. S13B). Finally, insulin has been reported to limit the initial specification of 2D cultured PrE-/VE-like cells termed nEND (Anderson et al., 2017; Zhong and Binas, 2019). Complementing the N2B27 medium with additional insulin or with the PI3K inhibitor ZSTK474 did not prevent Gata6^+^ cell specification and pro-amniotic-like cavity formation. However, additional insulin increased the overall size of EpiCs, consistent with a role in proliferation (Fig. S14). Differences between the 2D (Anderson et al., 2017) and 3D conformation might create additional layers of regulation of this pathway. Altogether, we concluded that, in chemically defined conditions, a restricted number of signaling pathways (GSK3β/β-catenin, Fgf, RA and cAMP) induces the specification of naive ESCs into PrE-like cells while maintaining Epi-like cells, and that a switch of signaling activity is necessary for the progression of the tissues, including a depletion of Lif for cavity formation and the putative presence of PI3K activators for growth. These data suggest that these two cell types provide each other with sufficient signals to support the morphogenetic transition.

### Nodal signaling from the Epi is required for the VE/Epi bonding

In the early post-implantation embryo, the absence of Nodal signals originating from the Epi prevents the acquisition of an embryonic VE identity and incomplete adherence between the VE and Epi (Mesnard et al., 2006; Brennan et al., 2001). Likewise, EpiCs using a Nodal homozygous knockout ESCs showed an increased level of disorganization where the VE layer partly delaminated and separated from the Epi compartment (Fig. 3I,L). In addition, laminin staining appeared irregular and scattered around the VE cells that produce it, thus possibly preventing the proper deposition of a continuous basal lamina onto the Epi (Fig. 3I). Concomitantly, Epi pro-amniotic-like cavities, marked by F-actin and Podxl, were often not evident, reinforcing the importance of Epi adhesion to the basal lamina for the establishment of Epi apical-basal polarity. However, XEn specification, marked by Gata6 and Pdgfrα expression, was not significantly affected (Fig. S15). Moreover, we ran a small screen using soluble factors on 72 h EpiC structures (Fig. S16) and observed that inhibition of Nodal/activin signaling using SB431542 showed a similar response to that observed in the Nodal double knockout line (Fig. 3I,J). Of note, both the Epi and VE tissues express β1 integrins and, as previously shown, embryos and EBs deficient for β1 integrins also display Epi/PrE delamination (Moore et al., 2014). Complementing the initial findings that Tgfβ superfamily signals originating from the Epi regulate VE development (Fig. 1G), these data suggest that Nodal signaling instructs the transition between PrE and VE. This Tgfβ signaling might directly induce the production of the basal lamina that serves as a base for epithelialization, via β1 integrin (Moore et al., 2014); they also suggest that Epi induction at the peri-implantation stage is important for the formation of an abutting double epithelium of VE and Epi tissues. Altogether, this suggests the existence of a two-way circuit regulating the co-development of these two tissues.

### EpiCs support both epiblast and visceral endoderm maturation

**Fig. 4.**
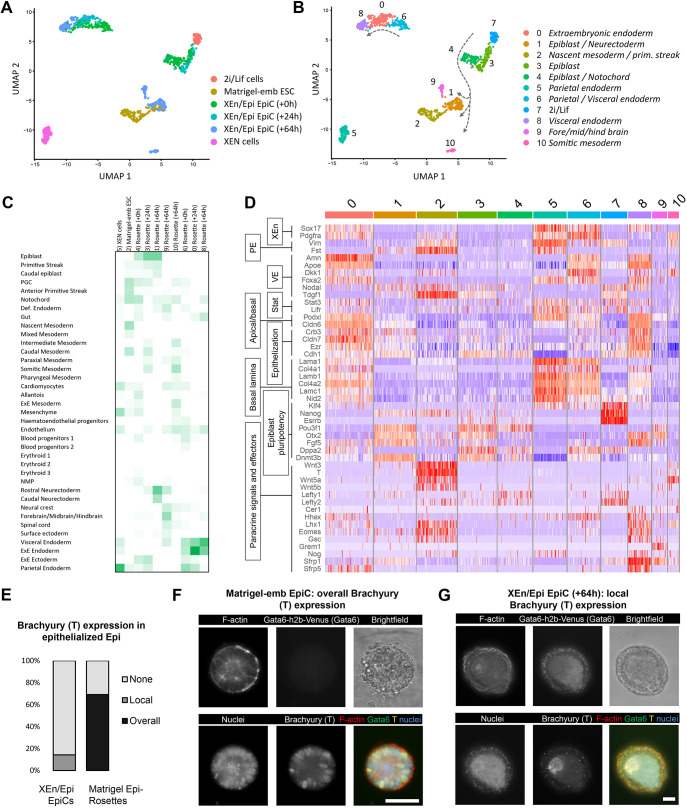
**Cellular identities of XEn/Epi EpiCs over time.** (A) UMAP plot of single-cell RNA-seq data of indicated culture conditions. Timepoints indicate the number of hours after EpiCs were flushed out from the microwells. Matrigel-emb ESCs were cultured for 96 h in total. XEN and 2i/Lif cells were cultured as monolayers. (B) Cell points are numbered and colored based on their computationally assigned cluster, and annotated by tissue type. Lines with arrows indicate the trajectory over time of EpiCs (+0 till +64 h). (C) Inferred tissue types per cluster by comparing top gene list with embryo data from Pijuan-Sala et al. (2019). (D) Heatmap plot depicting differentially expressed genes for extra-embryonic endoderm (XEn), parietal endoderm (PE), visceral endoderm (VE), STAT signaling, apical/basal polarity, epithelialization and basal lamina formation, epiblast pluripotency, and paracrine signals and effectors in the Nodal, BMP and Wnt pathways. (E) Brachyury (T) immunofluorescence found in epithelial-like epiblast compartments in XEn/Epi EpiCs (24 structures total) and in Matrigel-embedded Epi-EpiCs (13 structures total). (F,G) Representative immunofluorescence images of overall and local brachyury expression in (F) Matrigel-embedded Epi-EpiCs and (G) XEn/Epi rosettes (+64 h), respectively. Scale bars: 50 µm.

To more finely assess the state and reflected stage of the cells within the fully developed EpiCs, we performed additional single-cell RNA sequencing after 0, 24 and 64 h in plain N2B27 medium. We also included controls in the form of naive ESCs (2i/Lif), which are XEN cells that are thought to best reflect the parietal endoderm (Lin et al., 2016; Zhong and Binas, 2019), and of Matrigel-embedded ESCs, which form rosette-like cells undergoing lumenogenesis in the presence of serum but in the absence of XEn-like cells (Bedzhov and Zernicka-Goetz, 2014). We visualized single-cell distribution using uniform manifold approximation and projection (UMAP), and identified 10 distinct clusters (Fig. 4A,B). The top differentially expressed genes within each cluster was compared with expression maps of mouse gastrulation and early organogenesis (Pijuan-Sala et al., 2019) (Fig. 4C). We observed that the XEn compartment transitioned from a mixed parietal/visceral endoderm identity at 0 h (see also Fig. 2G,H) towards a more constrained VE identity at 64 h (*Amn^+^*/*Dab2^+^*, *Fst^−^*/*Afp^−^*; Fig. 4D, Fig. S17A). We concluded that the sustained contact with the Epi reduced the initial VE/PE heterogeneity and channeled the VE transcriptome. This transition was marked by initial Epi expression of known VE regulators *Nodal* and *Tdgf1* (*Crypto*) (Kimura et al., 2001; Kruithof-de Julio et al., 2011) and by the expression of genes involved in the STAT pathway (*Lifr* and *Stat3*), in epithelialization (*Crb3*, *Podxl*, *Cdh1*, *Cldn6/7* and *Ezr*) possibly initiated by *Foxa2* (Burtscher and Lickert, 2009), and in the deposition of extracellular basal lamina proteins (*Col4a1/2*, *Lmna*, *Lama1/b1/c1*, *Dag1* and *Nid2*; Fig. 4D, Fig. S17B). Consistent with an inductive role of Nodal in VE epithelialization, genes related to apical/basal polarity (*Podxl* and *Crb3*) and epithelial cells (*Cdh1*, *Cldn6*, *Cldn7* and *Ezr*) became progressively more prominently expressed over time. XEN cells clustered apart from the VE-like clusters, a distance that might reflect their PE identity (Fig. 4A,B, Fig. S17A). The Epi compartments at 0 and 24 h clustered largely together and expressed genes reflecting an early post-implantation Rosette-like identity (e.g. *Otx2*, *Fgf5*, *Oct6* and *Pou3f1*; Fig. 4D). The exit from naive pluripotency requires a transient downregulation of Wnt activity, possibly mediated by the Wnt inhibitor Dkk1 expressed by the PrE (Neagu et al., 2020). Accordingly, we observed a transient expression of Dkk1 in the PrE-like tissue (0 h) and its disappearance from 24 h onwards. The transience of Wnt inhibition would also subsequently allow the Epi to become receptive to autocrine and VE-secreted Wnt signals at gastrulation stage (Arnold and Robertson, 2009).

At 64 h, the Epi compartment spread into three different clusters with respective top-expressed genes found in native fore-, mid- and hindbrain, in Epi and neurectoderm tissues, and in somitic mesoderm. The first two clusters concomitantly expressed these germ layer-related genes along with post-implantation Epi-specific genes [*Klf4*^−^, *Oct6*^+^ (*Pou3f1*^+^) and *Otx2*^+^], suggesting a partial early anterior identity (Fig. 4D, Fig. S17C). The cluster expressing somitic mesoderm genes no longer expressed these Epi genes but was characterized by the expression of Wnt3 and Wnt5, which mark the posterior domain and are important for the gastrulation processes (Tortelote et al., 2013; Minegishi et al., 2017). When staining for brachyury (T), 14% of EpiCs (>64 h) contained brachyury-positive inner cells originating from an epithelium-like tissue (Fig. 4E-G). Additionally, the minority (20%) of structures that formed non-epithelized amorphous cell clumps, but included non-delaminated XEn layers, were all brachyury positive (Fig. S18). Of note, these amorphous structures were not included for single-cell transcriptomic analysis. To more finely assess the inductive role of the VE-like cells on the progression of the Epi-like cells, we compared the transcriptome with rosette-like cells embedded into Matrigel and cultured with serum (Bedzhov and Zernicka-Goetz, 2014). Most of these cells (96 h) clustered with the subpopulation reflecting a nascent mesoderm and/or primitive streak identity, while also partially overlapping with the Epi and neurectoderm (Fig. 4A,B). In comparison, Epi-like cells from the EpiCs also formed partial anterior-like cells (cluster 9, Fig. 4A,B,D, Fig. S17C). Consistent with a role for Epi epithelialization in facilitating the formation of the anterior Epi (Girgin et al., 2021), this suggests that the basal lamina regulates the formation of the posterior pre-gastrulation Epi, while additional signals originating from the XEn, possibly regulated by *Dkk1*, *Otx2*, *Lhx1* and *Foxa2* (Perea-Gomez et al., 2001), are conductive for the formation of the anterior cells. Overall, we concluded that reciprocal interactions between the Epi and VE are sufficient to initiate a program that reflects the formation of anterior and of posterior, gastrulating, Epi.

Induction of gene expression that originates from the trophoblasts, including *Bmp4*, regulates the expression of Wnt ligands and gastrulation (Rivera-Pérez and Magnuson, 2005). Although we observed Wnt3 and Wnt5 expression in the Epi-like tissue, we did not detect Wnt3 expression in the VE-like tissue, which is known to be produced first during development (Arnold and Robertson, 2009). In addition, trophoblast-secreted BMP4 maintains Nodal levels in the Epi first locally via a SMAD, which is a FoXH1 autoregulatory enhancer, and then through the activation of an autoregulatory posterior Wnt3 loop. Here, Nodal was initially not expressed in the VE and was expressed at low levels in the Epi (0/24 h), likely due to the absence of trophoblast signals; however, its expression later increased in both the VE- and Epi-like tissues (64 h). This suggests an alternative induction route separate from BMP and Wnt signals. Altogether, these data suggest that, although trophoblastic tissues are important for the anterior-posterior patterning of the Epi (Stephenson et al., 2012), Epi-VE interactions are sufficient to initiate part of the gastrulation program, including the expression of Wnt ligands in the Epi and Wnt inhibitors in the VE.

The VE is known to form a subpopulation of anterior VE that migrates toward the prospective anterior Epi, which, under the control of Foxa2 (Kimura-Yoshida et al., 2007) and Otx2 (Perea-Gomez et al., 2001), secretes inhibitors of the Wnt and Nodal signaling pathways to facilitate formation of the anterior tissues (Kimura-Yoshida et al., 2005; Arnold and Robertson, 2009). Accordingly, the 64 h VE-like tissue expressed *Foxa2*, *Otx2*, the Wnt ligand inhibitor *Sfrp1*, Wnt agonists *Hhex* and *Sfrp5*, and modulators of Nodal activity (*Gsc*, *Eomes* and *Lhx1*), but barely expressed the ensuing factors *Cer1*, *Dkk1*, *Tdgf1* (*Crypto*) and *Lefty1* (Fig. 4D, Fig. S17D). We concluded that, as previously observed (Rodriguez et al., 2005), the Epi/VE interaction is sufficient to promote the expression of some DVE genes regulating the expression of inhibitors, including *Sfrp1* and *Gsc*, but that is insufficient to regulate anterior Epi effector genes such as *Cer1*, *Dkk1*, *Tdgf1* (*Crypto*), *Lefty1*, *Spp1*, *Zbp1* and *Aire* (Cheng et al., 2019). Accordingly, microdissection of the ExE of E5.5 concepti showed that this tissue represses the expression of *Cer1* and *Lhx1* in the DVE (Rodriguez et al., 2005). EpiCs might be excluded from the element of the Epi/VE interaction that regulates *Cer1* and *Lhx1*, or there could be an earlier unreported role of the trophoblasts in inducing the formation of the VE and/or DVE. Altogether, we concluded that supervision of the DVE by its interaction with both the Epi and the trophoblast might ensure the expression of Wnt and Nodal inhibitors.

### In blastoids, the four molecules prime ESCs to form primitive endoderm-like cells

**Fig. 5.**
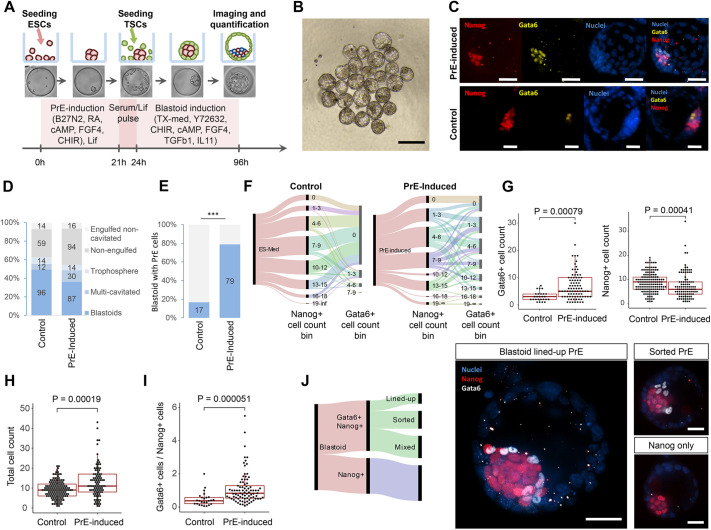
**The PrE/Epi priming of ESCs induces the formation of the niche in blastoids.** (A) Schematic for PrE-induced blastoid formation. (B) Bright-field image of representative selection of PrE-induced blastoids. Structures with a single cavity and an inner cell compartment were classified as blastoids. (C) Maximum intensity confocal projection immunofluorescence images of representative PrE-induced and control blastoids stained for Nanog (red) and Gata6 (yellow). DAPI staining (blue) shows cell nuclei. Scale bars: 200 µm. (D) Percentages of the different structures found in microwell arrays in control (*n*=195) and PrE-induced (*n*=241) conditions, pooled from two wells per condition. (E) Percentage of blastoids including Gata6^+^/Nanog^+^ cells formed under the control of PrE-induced conditions (left). Pooled results from three datasets. ***P<0.001, Fisher's exact test. (F) Alluvial diagram displaying cell count of Nanog^+^ and Gata6^+^ dichotomy for control and PrE-induced blastoids (left). (G) Gata6^+^ and Nanog^+^ cell counts compared between control and PrE-induced blastoids that contain both Nanog^+^ and Gata6^+^ cells. (H) Total number of inner cells within blastoids. (I) Ratio of Gata6^+^/Nanog^+^ cells per blastoid containing both Gata6^+^ and Nanog^+^ cells. Boxes and whiskers indicate the first, median and third quartile, and minimum and maximum data points excluding outliers, respectively. (J) Alluvial diagram displaying contributions of resulting phenotypes following PrE-induced blastoid formation. Representative immunofluorescence images of PrE/Epi blastoid phenotypes. Scale bars: 50 µm. Data in D-I are derived from two independent experiments with three pooled wells each. In G-I, the *P*-values were determined using the Mann–Whitney *U*-test.

Next, we tried to enhance the formation of PrE-like cells in blastoids to eventually model the impact of the two extra-embryonic tissues on Epi development. We thus modified the original blastoid protocol (Rivron et al., 2018a) by exposing ESCs, including a fluorescent reporter for Gata6 (ESCs^Gata6-h2b-venus/+^) (Freyer et al., 2015), to the inductive molecules during the aggregation phase (0–24 h, Fig. 5A), i.e. before adding the TSCs. PrE-induction tempered the efficiency of blastoid formation (from 49% to 36%, specified as a single trophoblast cavity enveloping ESCs, Fig. 5B) by reducing the efficiency of TSCs to engulf the EBs (from 39% to 30% of non-engulfed structures, Fig. 5B). Although the underlying reasons are unknown, the specification of PrE-like cells might coincide with a change in their surface properties, reducing the capacity for TSCs to englobe them. Nevertheless, the molecules increased the overall percentage of blastoids, including Gata6^+^ and Nanog^+^ cells from 22% to 78% (Fig. 5C-F, Fig. S19). Concomitantly, the number of Gata6^+^ cells increased (*P*=0.00079, Fig. 5E,G). Notably, the total number of Epi plus PrE cells also increased (Fig. 5H) and the ratio of Gata6^+^ to Nanog^+^ cells in PrE-induced blastoids (Fig. 5I) was comparable with the one in blastocysts (0.83 versus 0.9 in 120 cells-stage blastocysts (Saiz et al., 2016). In accordance with our observations in EpiCs and with a previous study (Saiz et al., 2016; Nowotschin et al., 2019; Hiramatsu et al., 2013), PrE and Epi cells co-regulate their specification and proliferation.

Next, we examined the spatial organization of PrE-induced blastoids and observed that 21% of the blastoids, including PrE-like cells, showed a layer of Gata6^+^ cells lined up along the cavity of the blastoid (Fig. 5J), similar to E4.5 blastocysts (Hermitte and Chazaud, 2014; Ohnishi et al., 2014). Among the other blastoids, 35% comprised sorted but not aligned Gata6^+^ cells, while 44% had the salt and pepper phenotype of Gata6^+^ and Nanog^+^ cells (Fig. 5J) that is reminiscent of an earlier blastocyst stage (Frankenberg et al., 2011; Plusa et al., 2008; Meilhac et al., 2009).

### In blastoids, the expansion of the primitive endoderm-like tissue supports the formation of post-implantation-like structures *in vitro*

**Fig. 6.**
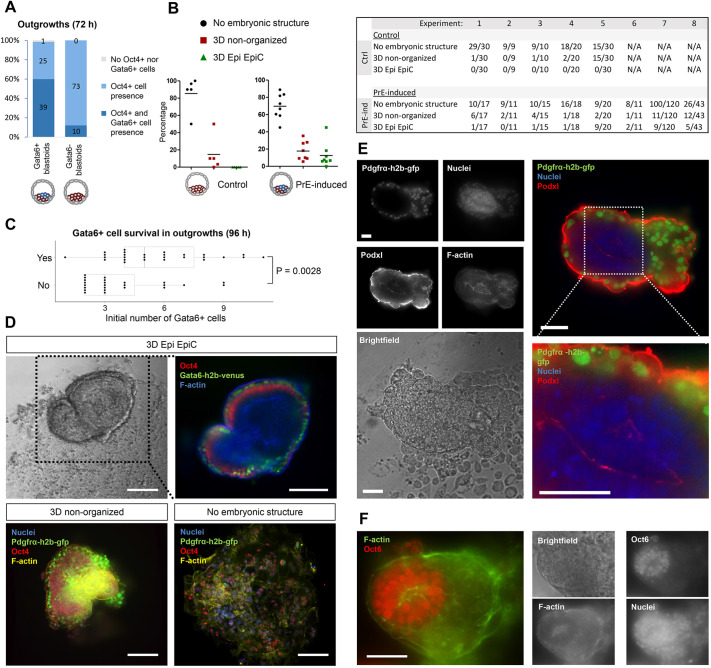
**The induction of the PrE-/Epi-like niche in blastoids supports the formation of post-implantation-like tissues.** (A) Presence of Epi (Oct4^+^) and PrE (Gata6^+^) cells within *in vitro* outgrown (for 72 h) PrE-induced blastoids with and without Gata6^+^ cells (96 h). Total number of structures pooled from four experiments are displayed within the bars. (B) Percentage (left) and number (right) of different tissue phenotypes arising from PrE-induced blastoids including two or more Gata6^+^ cells compared with non-induced blastoids. Every data point represents an independent experiment. Monolayer outgrowths were classified as ‘no embryonic structure’; structures with 3D outgrowths without a pro-amniotic-like cavity and irrespective of cell type were classified as ‘3D non-organized’. Structures with 3D outgrowths that contained Epi cells, PrE cells and had a pro-amniotic-like cavity, as observed by F-actin and/or Podxl staining, were classified as ‘3D Epi EpiC’. (C) The presence of PrE tissue (Gata6^+^) within *in vitro* grown PrE-induced blastoids (yes/no, at 96 h) as a function of the numbers of Gata6^+^ cells within the initial blastoids. Each point represents an individual cell aggregate. *P*-values were determined by a Mann–Whitney *U*-test. (D) Top: bright-field and immunofluorescence images of an *in vitro* grown blastoid with Oct4^+^ Epi (red) and Gata6^+^ PrE (green) cells surrounding a pro-amniotic cavity and growing on top of a TSC monolayer (96 h). Bottom: representative images of *in vitro* grown blastoid phenotypes with Oct4^+^ Epi (red), Pdgfrα^+^ PrE (green) and overall F-actin (yellow) and nuclei (blue). Scale bars: 200 µm. (E) Pdgfrα^+^ cells surrounding an epiblast-like tissue, including a pro-amniotic-like cavity marked by Podxl expression (72 h). Scale bars: 50 µm. (F) Oct6^+^ epiblast-like tissue blastoid outgrowth (48 h). Scale bar: 50 µm.

Finally, we tested whether the PrE/Epi-like tissues within blastoids could support the formation of tissues reflecting the post-implantation stage. We cultured PrE-induced blastoids containing PrE cells (>2 Gata6^+^ or Pdgfrα^+^ cells) and non-induced blastoids *in vitro* (Bedzhov et al., 2014; Hsu et al., 1974). Induction of the ESCs at the onset of blastoid formation did not affect the final presence of Epi cells (96 h, 98% versus 100%, Fig. 6A) but enhanced the potential of the PrE-like cells to expand (96 h, Gata6^+^, 60% versus 10%, Fig. 6A). This effect correlated with the initial number of PrE cells present in blastoids and is reminiscent of the FGF4 induction of PrE in blastocysts (Fig. 6C). The presence of the PrE-like cells did not improve the formation of non-organized 3D structures (experimental average of 18% versus 15%, Fig. 6B; pooled yields of 15% versus 19% from eight independent experiments) containing both Oct4^+^ Epi and PrE cells (Fig. 6C, Fig. S20) but endowed some blastoids with the capacity to support the formation of EpiCs marked by Podxl expression (Fig. 6D, Fig. S20) (11% of blastoids, pooled yield in eight independent experiments; Fig. 6C), the Epi of which transitioned into a Oct6^+^ post-implantation-like state (Fig. 6F); however, ExE-like tissue formation appeared absent. The non-induced blastoids lacked that potential (Fig. 6C). We concluded that a threshold in the number of PrE-like cells is crucial to support the progression of the post-implantation Epi-like tissue in blastoids.

**Fig. 7.**
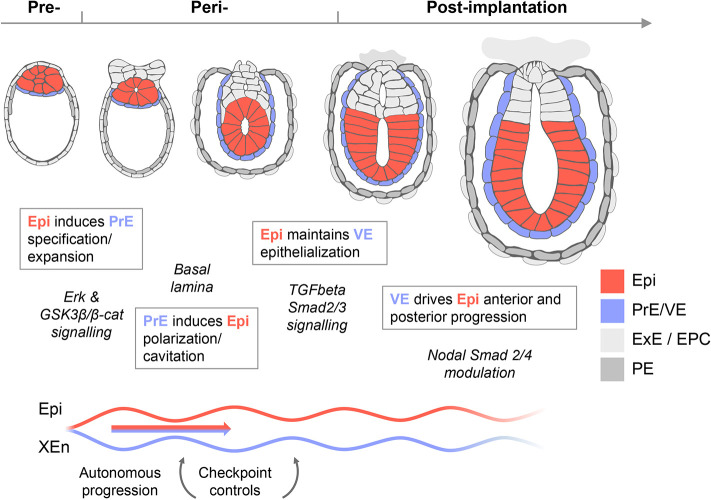
**Self-organized reciprocal inductions and pathways underpinning the co-developing XEn-/Epi-like tissues.** Signals from the pre-implantation Epi (Erk and GSK3β/β-catenin signaling) support the primitive endoderm (PrE) to produce a basal lamina that subsequently regulates Epi polarization and cavitation. In exchange, the Epi channels the transcriptomic progression to VE through TGFβ signals. This VE then contributes to Epi bifurcation into anterior- and posterior-like states. In this model, self-organization arises from lineage bifurcation followed by a pendulum of induction that propagates over time.

Altogether, we concluded that the Epi/XEn tissues are sufficient to support aspects of specification and proliferation to the rosette and lumenogenesis stages through the deposition of a basal lamina (Fig. 7). During the early post-implantation stage, the Epi supports further progression of the VE through the secretion of Nodal and Tdgf1 that facilitates the progression of two abutting VE/Epi epithelia. At the post-implantation/pre-gastrulation stages, the VE not only provides structural support through the formation of a basal lamina, but also appears to produce additional signals that contribute to the formation of both the anterior (e.g. *Lhx1*, *Otx2* and *Foxa2*) and posterior Epi.

## DISCUSSION

High-content screening of a large number of EBs on a microwell array in chemically defined culture conditions allows for robust statistics necessary to delineate the effect of signaling pathways. Here, we observed that the combination of FGF4, Wnt, cAMP and RA is sufficient to rapidly and efficiently drive the co-formation of PrE- and Epi-like cells in gel-free and serum-free 3D cultures.

Upon transfer into plain N2B27 medium, these two cell types are capable of further growth and autonomous organization into a structure that undergoes aspects of post-implantation development. We found that the PrE cells in PrE/Epi-induced structures before forming a pro-amniotic cavity show an initial bifurcation into PE and VE precursors independently from trophoblastic tissues (Fig. 2F-H), suggesting this to be a cell-autonomous mechanism that could be stochastic or partly tuned by signals from the Epi. However, when Epi/PrE-like structures underwent rosette morphogenesis, the identity of PrE tissues was channeled into a VE-like identity, not a PE identity, suggesting that the PE-precursors are lost or transcriptionally normalized over time by contact with the Epi. This suggests the possibility that the cells can fluctuate between states at the time of specification, followed by a channeling of the state due to an inductive environment. This initial fluctuation could endow the embryo with adaptive and regulative capacities (Paca et al., 2012).

The single-cell RNA-sequencing data suggest an early peri-implantation Epi identity for the embryonic clusters in 0/24 h EpiCs (Fig. 4). In contrast, embryonic clusters in 64 h EpiCs showed four distinct clusters that, although they clearly express Epi markers (Oct4 and Otx2), appear already transcriptionally primed for mesodermal and ectodermal progression. Notably, a definitive endoderm cluster was not found, possibly due to low combined levels of activin/Nodal and the BMP inhibitor noggin (Fig. S16D), which suggests the requirement of additional supporting tissues (e.g. trophoblastic). Of note, XEN cells clustered separately, with a seemingly parietal endoderm identity.

Consistent with the idea that blastocysts are self-regulating systems adjusting the ratios and numbers of their three founding cell types (Saiz et al., 2016), the induction of PrE-like cells in blastoids triggers a regulation of the total cell number. The *in vitro* culture of blastoids (Hsu, 1971) additionally suggests that a threshold of PrE cells is necessary to sustain the expansion and morphogenetic capability of the Epi during the post-implantation stage. Accordingly, the insufficient formation of PrE and incomplete lining of the PrE epithelium between the Epi and blastocoel, e.g. in GATA6 KO conceptus, has been described to halt Epi expansion in blastocysts (Artus et al., 2010; Moore et al., 2014). Similarly, inappropriate specification of the extra-embryonic VE results in disorganized ectoderm and stagnated development (Barbacci et al., 1999).

Overall, these results argue, beyond the inductions originating from the Epi (e.g. FGF4), for the importance of the PrE tissue in supporting the Epi for survival and expansion. The mechanisms by which the PrE accomplishes this remain to be determined. One attractive possibility is that Epi proliferation and morphogenesis needed to form the amniotic cavity cannot occur unless the PrE deposits the required basal lamina, similar to laminin-KO embryos that exhibit aberrant peri-implantation morphogenesis and halted development (Miner and Yurchenco, 2004; Smyth et al., 1999). It delineates a critical switch of signaling activity necessary to transit from pre- to post-implantation development, and suggests that the deposition of a functional amount of basal lamina acts as a checkpoint for developmental progression. During post-implantation development, the VE appears to play an additional role, beyond the maintenance of the basal lamina that is controlled by Nodal originating from the Epi, to drive the progression of the different tissues emerging from the Epi. Considering that blastoids do not progress upon implantation *in utero*, it would be interesting to find out in future studies whether PrE-induced blastoids have an improved capacity to do so. Altogether, this study contributes to the establishment of stem cell-based embryo models amenable to high-throughput drug and genetic screens, which may alleviate the burden on animal use (Andersson-Rolf et al., 2017; Rivron et al., 2018b) and become a foundation for basic and biomedical discoveries to elucidate the crucial and currently unknown processes of embryogenesis.

## MATERIALS AND METHODS

### Microfabrication

Elastomeric stamps for imprinting the agarose microwell arrays were fabricated using PDMS Sylgard 184 kit. Microwell arrays were molded as described previously (Vrij et al., 2016b) using a 2.2% w/v solution of Ultrapure agarose (ThermoFisher, 11560166). Each well of a 96-well plate with 430 microwells of 200 µm was molded. Each well contained a calculated liquid volume of 250 µl split between 225 µl medium and 25 µl hydrogel buffer.

### Cell culture

The following lines were used for experiments: Pdgfrα h2b-gfp/+, h2b-rfp V6.5 sub-clone, Gata6-h2b-Venus/+;ColA1 TetO-Gata4-mCherry/+;R26 M2rtTA/+ ES cells and V6.5 Nodal KO (−/−) with corresponding wild types. The Gata6-h2b-Venus cell line was a kind gift from C. Schröters’ laboratory (Max Planck Institute of Molecular Physiology, Dortmund, Germany; Freyer et al., 2015). The V6.5 cell line has a C57BL/6×129/Sv background and was obtained from the laboratory of R. Jaenisch (Whitehead Institute for Biomedical Research, Cambridge, MA, USA). The Pdgfrα h2b-gfp/+ cell line has an ICR background and was derived in the laboratory of A.-K. Hadjantonakis (Sloan Ketttering Institute, New York, USA).

ESCs were seeded as 25,000 per cm^2^ and expanded on 0.1% w/v gelatin-coated tissue culture-treated polystyrene dishes (Nunc). Cell expansion medium was carried out in N2B27 2i/Lif conditions comprising 2% B27 (Gibco, 17504-044) and 1% N2 (Gibco, 17502-048) in a 1/1 mixture of DMEM/F12 (ThermoFisher, 11039021) or Advanced DMEM/F12 (ThermoFisher, 12634028) with Neurobasal (ThermoFisher, 12348017), including 2 mM Glutamax (Gibco), 10 mM NEAA (ThermoFisher, 11140050), 0.5% bovine serum albumin (Sigma-Aldrich, A7979), 10 mM HEPES (Gibco, 15630056), 1 mM sodium pyruvate (ThermoFisher, 11360070), supplemented immediately before use with 10 ng/ml leukemia inhibitory factor (Lif, Merck Millipore ESG1106), 1 μM PD0325901 (AxonMed, 1408), 3 μM CHIR99021 (AxonMed, 1386) and 50 μM 2-mercaptoethanol (Gibco, 11528926), as developed previously (Nichols et al., 2009).

ESCs were expanded for a minimum of two passages before aggregating into EBs within the agarose microwells. For cell banking and serum/Lif experiments, ESCs were expanded on a monolayer of mouse embryonic fibroblasts (mEF) in serum-medium consisting of DMEM containing sodium pyruvate and Glutamax (Thermo Fisher, 10569010) supplemented with 10% serum, 10 mM NEAA, 10 mM HEPES, 10 ng/ml Lif and 50 μM 2-mercaptoethanol.

TSCs were seeded and expanded as 25,000 per cm^2^ on 3% Matrigel-coated dishes in chemically defined TX-medium, as developed previously (Kubaczka et al., 2014) or on TCPS with a Laminin-512 coating (Biolamina LN521-02, 5 µg/ml overnight incubation at 4°C) in TX-medium supplemented with activin A (Bio-techne, 338-AC-010), 50 ng/ml IL11 (Peprotech, 220-11), 200 µM 8Br-cAMP (Biolog, B007-500), 25 ng/ml BMP7 (R&D Systems, 5666-BP-010), 5 nM LPA (Tocris, 3854), 2 ng/ml TGFβ1 (Peprotech, 100-21), 25 ng/ml FGF4 (R&D Systems, 5846-F4-025 and 7486-F4), 1 µg/ml heparin (Sigma-Aldrich, H3149) and 100 µM 2-mercaptoethanol. Extra-embryonic endoderm (XEN) cells were expanded on 0.15% gelatine-coated TCPS culture plates in RPMI 1640 medium (ThermoFisher, 11875085), including L-glutamine, supplemented with 20% FBS (fetal bovine serum, embryonic stem cell-qualified, ThermoFisher, 16141061), 1 mM sodium pyruvate, 10 mM HEPES, 100 µM 2-mercaptoethanol. Y27632 (AxonMed, 1683; 0.5 µM) was added to the medium when cells were passaged. All cells were routinely checked for mycoplasma infection.

### EB, PrE/Epi and XEn/Epi formation

EBs were formed by seeding an average of seven ESCs per microwell in either serum-containing medium, Lif/serum-containing medium or N2B27-based media, all supplemented with 50 µM of 2-mercaptoethanol. Lif/serum-containing medium consisted of DMEM containing sodium pyruvate and glutamax (Thermo Fisher, 10569010) supplemented with 10% serum, 10 ng/ml Lif, 10 mM NEAA, 10 mM HEPES and 100 U/ml penicillin/streptomycin. PrE-induction medium consisted of advanced N2B27 medium supplemented with 3 μM CHIR99021, 50-100 ng/ml FGF4, 10 nM RA, 1 mM 8Br-cAMP and 50 µM 2-mercaptoethanol. To induce XEn/Epi EpiCs from PrE-induced EBs, 14 ESCs were seeded per microwell supplemented with 2 µM Y27632 (AxonMed 1683). Occasionally, microwell arrays were pre-wetted in serum-medium containing 100 U/ml penicillin/streptomycin before use. For XEn/Epi EpiC formation using the Nodal double KO (−/−) line, 10% of serum was added to the medium. After 24 h or 48 h of culture, cells were washed once with advanced N2B27 medium and then refreshed with advanced N2B27 supplemented with 50 µM 2-mercaptoethanol. After 72 h, EBs were flushed out and transferred into non-TCPS six-well plates with 2 ml advanced N2B27 medium supplemented with 50 µM 2-mercaptoethanol. Then, after an additional 48 h of culture, structures were either fixated using a fresh solution in PBS of 2% formaldehyde and 0.1% glutaraldehyde, or half of the medium was refreshed and structures were cultured for an additional 24 h before fixation.

Epiblast-only rosettes (used as controls in the scRNAseq data) were formed by first seeding eight ESCs per microwell in serum-containing medium. After 12 h, the ESC aggregates were flushed out and resuspended in 15 µl Matrigel droplets cultured in serum-containing medium.

### Blastoid formation

Blastoids were formed as described previously (Rivron et al., 2018a; Rivron, 2018). For control blastoids, an average of seven ESCs were seeded per microwell in serum/Lif medium (10 ng/ml Lif, control blastoids) with 50 µM 2-mercaptoethanol. For PrE-induced blastoids, an average of seven ESCs were seeded per microwell in either (1) N2B27 medium with PrE-induction compounds and 10 ng/ml Lif for 21 h incubation followed by serum/Lif for 3 h, or (2) serum/Lif medium with PrE-induction compounds. After 24 h of ESC aggregation an average of 17 TSC were added per microwell in TX medium with non-essential amino acids and the blastoid culture components (20 µM Y27632, 5 µM CHIR99021, 1 mM 8Br-cAMP, 25 nG/ml FGF4, 2 nG/ml TGFβ1, 30 nG/ml IL11, 1 µg/ml heparin and 100 µM 2-mercaptoethanol). In some experiments, after 24 h an additional 1 mM of 8Br-cAMP was added to the blastoid culture medium. Structures with a single cavity and an inner cell compartment were classified as blastoids. Full stacks using a spinning disk confocal with a 40× objective were made for the counting of PrE and Epi cells within blastoids (Fig. 5). The fluorescence images of seven blastoids in Fig. 5 were made by stacking three to five spinning disk confocal slides.

### *In vitro* post-implantation assay

Blastoids cultured for 96 h (from the time of seeding ESC) were selected based on their morphology (cystic, roundness, presence of inner cell mass) and transferred from microwells onto tissue-culture glass or polystyrene plastic in IVC1 medium using mouth pipetting. IVC1 medium consisted of Advanced DMEM/F12 medium with non-essential amino acids and sodium pyruvate, 20% ESC-selected fetal bovine serum, 2 mM glutamax, penicillin/streptomycin, 1/100 ITS-X (ThermoFisher, 51500056), 8 nM β-estradiol (Sigma-Aldrich, E8875), 200 ng/ml progesterone (Sigma, P8783), 25 µM Acetyl-l-cysteine (Sigma-Aldrich, A9165-5G) and 50 µM 2-mercaptoethanol. Structures were fixated after 72 or 96 h of culture using a fresh solution in PBS of 2% formaldehyde and 0.1% glutaraldehyde.

### Microscopy and image-based analysis

For the soluble factor screen, the fluorescence images were acquired in widefield on a widefield Nikon Eclipse Ti microscope using a 10×objective, Andor Zyla4.2 camera and a Lumencor SpectraX as light source with LED lines of 390, 490, 568 and 647 nm that were used in this study. The standard DAPI (DAPI-5060C), GFP (GFP-3035D-000), Txred (TXRED-4040C0360), Cy5 (Cy5-4040C) and Cy7 (Cy7-B) filter cubes from Semrock were used. Analysis of EBs was performed using a custom-made pipeline in CellProfiler2.0 or 3.1 (Broad Institute) (Lamprecht et al., 2007). The number of EBs and cells positive for Pdgfrα-h2b-gfp or Gata6-h2b-venus were determined by thresholding on intensity. EBs were considered positive for Pdgfrα when one or more cells were positive for Pdgfrα-h2b-gfp. The number of Pdgfrα^+^ cells was identified from widefield images acquired within the equatorial plane of EBs, and therefore reflect a proxy for the total number of Pdgfrα^+^ cells per EB. Mouse blastocyst images were deconvolved using Huygens Professional software and quantified using Imaris x64 9.5.0.

### Soluble factor screening

Serial dilutions of compounds were made in appropriate solvents (DMSO or H_2_O) and corresponding carrier controls were included in the assays. Serial dilutions were made for single soluble factor titrations; FGF4, sodium (ortho)vanadate (Sigma-Aldrich, S6508), PD0325901, PD98059 (Sigma, P215), activin A, TGFβ1, A83-01 (Tocris, 2939), Nodal (R&D systems, 1315-ND-025), SB431542 (Tocris, 1614), retinoic acid (Sigma-Aldrich, R2625), DL-epinephrine HCl (Sigma-Aldrich, E4642), 8Br-cAMP, SC144 (Tocris, 4963/10), IL6 (Peprotech, 216-16), IL11, Lif, BMP4 (Peprotech, 315-27), BMP7, LDN 193189 (Tocris, 6053), ML347 (Selleckchem, S7148), Noggin (Peprotech, 250-38), CHIR99021, IWP2 (Selleckchem, S7085) and XAV-939 (Selleckchem, S1180). Compounds with end concentrations that were used for XEn/Epi EpiC modulation: PI3K inhibitor ZSTK474 (Selleckchem, S1072) at 1 μM, insulin at 50 ng/ml, XAV-939 at 20 μM.

### Mouse embryo culture

Embryos were flushed from the uterus as compacted morulas and incubated at 37°C in basic KSOM medium (Sigma-Aldrich, MR-101-D). Upon cavity formation, the embryos were transferred to KSOM medium supplemented as listed in Fig. S9 and incubated in the respective media at 37°C. Control medium was KSOM only. After 42-48 h blastocysts were fixed in 4% formaldehyde in PBS. If they had not hatched or were in the process of hatching, the zona pellucida was removed before fixation using Tyrode's solution.

### Immunofluorescence

Blastoids and blastocysts were fixed in 4% formaldehyde solution in PBS for 15 min at room temperature. XEn/Epi EpiCs and *in vitro* implantation cultures were fixated in 2% formaldehyde solution with 0.1% glutaraldehyde in PBS for 15 min at room temperature. After fixation, samples were washed three times in washing buffer (0.1% Triton-X with 2% BSA in PBS), permeabilized in a 1% Triton-X solution in PBS and blocked in blocking buffer (2% BSA, 5% serum of host secondary antibody species, 0.5% glycine, 0.1% Triton-X, 0.2% Tween-20) for 30 min. Samples were incubated in antibody solution (25% blocking buffer with 50% PBS and 25% 0.1% Triton-X in PBS) with primary antibodies for 12 h at 4°C, washed three times for 10 min with washing buffer followed by incubation with secondary antibodies in antibody solution for 4 h at 4°C. Some samples were also stained with DAPI (0.2 µg/ml) and phalloidin (ThermoFisher, A22287 or A12380, 1/100 dilution). A list of used antibodies can be found in Table S4.

### Single-cell sequencing

EBs were washed twice in PBS before collagenase IV (600 U/ml) was added. Plates were shaken at 350 rpm for 30 min before being transferred to TrypLE 10X (Thermofisher, A1217701, no dilution) and shaken again for 20 min. EBs were dissociated into single cell suspensions using a small capillary. The resulting suspension was quenched using PBS with 50% FBS, centrifuged at 200 ***g*** and resuspended in 230 µl of PBS with 10% FBS. Cells were stained with Pdgfrɑ antibody (1:150 dilution) for 30 min at 4°C followed by three PBS (+10% FBS) washes and secondary antibody incubation (1:400) for 30 min. Cells were washed three times in PBS and sorted for further processing.

XEn/Epi EpiCs were manually picked and placed in a round-bottomed 96-well plate containing 100 µl PBS+0.5% BSA. When sufficient numbers were collected, they were transferred to an Eppendorf tube with 600 µl Accumax and incubated at 37°C for 30 min. Every 5-10 min the mixture was resuspended using a 200 µl pipette. When single cells were observed under the microscope, the suspension was centrifuged at 200 ***g*** for 4 min, supernatant was removed, and cells were resuspended in staining solution in PBS (2 µM Di-I+0.1% Dead-stain-647) and incubated for 20 min at room temperature in the dark.

Epiblast-only rosettes were washed once with PBS before domes were disrupted using 150 µl of a 1:1 DipaseII:N2B27 mixture and incubated for 20 min at 37°C in a 1.5 ml Eppendorf tube (four domes per tube). Solution was resuspended every 5-10 min using a 200 µl pipette tip. Finally, the cell suspension was resuspended in 700 µl Accumax with 100 µl of PBS with 0.5% BSA and incubated for 25 min at 37°C. Single cells were stained with 2 µM Di-I+0.1% Dead-stain-647 and incubated for 20 min at room temperature in the dark.

### Single-cell sequencing experiments

For single-cell sequencing, EBs, Matrigel-embedded Epi rosettes and XEn/Epi EpiCs were manually picked and incubated in AccuMax solution at 37°C for 30 min and resuspended every 5 to 10 min to aid single-cell dissociation. For Matrigel-embedded Epi rosettes, the basal lamina gel was first removed by one time washing with PBS followed by Cultrex organoid harvesting solution (R&D Systems, 3700-100-01) or a 1:1 mix of DispaseII:N2B27 medium and incubated for 25 min at room temperature or 37°C, respectively. Dissociated cells were stained for a live staining (Vybrant DiI Cell-Labeling Solution, ThermoFisher, V22885) and dead staining (LIVE/DEAD Fixable Near-IR Dead Cell Stain Kit, ThermoFisher, L34975) to improve selection of viable cells. Gata6-h2b-Venus^+^ cells, which are indicative of PrE/VE, and non-fluorescent single cells were sorted in 384-well-plates for further RNA sequencing.

### SORT-seq, sequencing and mapping of scRNA-seq data

The sorted 384-well plates were processed using the SORT-seq protocol (Muraro et al., 2016) and sequenced on an Illumina NextSeq500 sequencing platform yielding paired-end reads of 75 bp. The second sequencing run (associated with data in Fig. 4) was performed by SingleCellDiscoveries. The first six nucleotides of the read unique molecular identifier were followed by a unique cell barcode that was used to perform demultiplexing. After demultiplexing, read 2 was used to map to the mouse genome (mm10) using TopHat (v2.1.1) (Kim et al., 2013). The count table per single cell was obtained as previously described (Markodimitraki et al., 2020).

### scRNA-seq data processing

scRNA-seq data was processed using Seurat (v3) (Stuart et al., 2019). Cells with more than 1000 genes detected were selected and genes present in fewer than three cells were removed from the analysis. Expression was normalized to 10,000 transcripts and the 3500 most variable transcripts were used for downstream analysis. Data were then scaled to the total number of transcripts per cell. After principal component analysis, 20 principal components were used for downstream analysis. Clustering, t-distributed stochastic neighbor embedding (t-SNE) and uniform manifold approximation and projection (UMAP) were performed using Seurat default parameters.

### Data analysis and reproducibility

Sample sizes and statistical tests for every experiment are provided in the figure legends. Sample sizes were not predetermined using statistical methods. If not stated otherwise, all data are displayed as mean±s.d.

PrE-induced blastoid formation and *in vitro* implantation assays were repeated at least five times using two ESC and two TSC lines. Alluvial figures were created using RAW – an open source project by DensityDesign Lab and Calibro (Mauri et al., 2017).

Single-cell transcriptome analysis, including clustering and heatmaps, was performed in R (https://www.r-project.org/) using the Seurat package for R (https://satijalab.org/seurat/). A minimal detection threshold of 5000 genes per cell was selected for cells to be included for analysis. Gene ontology enrichment analysis was performed using GOrilla (Eden et al., 2009).

Bar plots were created using Microsoft Excel. SPRING Louvain clustering was performed using Kleintools (Weinreb et al., 2018). Scatter plots in Fig. 2 were generated using GraphPad Prism 5. Scatter plots in Fig. 4, violin plots and dose-response curves were generated using R with the packages ‘ggplot2’, ‘reshape2’, ‘ggsignif’ and ‘ggbeeswarm’. Statistical analysis was performed using the package ‘stats’.

## Supplementary Material

Reviewer comments
