# Supplementary material for: A pendulum of induction between the epiblast and extra-embryonic endoderm supports post-implantation progression
Source: Development. 2022 Aug 22;149(20):dev192310. doi: 10.1242/dev.192310 (PMC9534490; doi:10.1242/dev.192310)
Supplement: Reviewer comments [file develop-149-192310_review_history.pdf]

A pendulum of inductions between the epiblast and extra-embryonic endoderm supports post-implantation progression

Erik J. Vrij, Yvonne S. Scholte op Reimer, Laury Roa Fuentes, Isabel Misteli Guerreiro, Viktoria Holzmann, Javier Frias Aldeguer, Giovanni Sestini, Bon-Kyoung Koo, Jop Kind, Clemens A. van Blitterswijk and Nicolas C. Rivron
DOI: 10.1242/dev.192310

Editor: Matthias Lutolf

Review timeline
Original submission: 6 May 2020
Editorial decision: 2 June 2020
First revision received: 16 May 2022
Accepted: 23 June 2022

Original submission

First decision letter

MS ID#: DEVELOP/2020/192310

MS TITLE: Expansion of the extraembryonic endoderm in blastoids supports post-implantation morphogenesis of the epiblast.

AUTHORS: Erik Vrij, Yvonne Sholte Op Reimer, Javier Frias Aldeguer, Isabel Misteli Guerreiro, Bon-Kyoung Koo, Clemens van Blitterswijk, Jop Kind, and Nicolas Rivron

I have now received all the referees' reports on the above manuscript, and have reached a decision. The referees' comments are appended below, or you can access them online: please go to BenchPress and click on the 'Manuscripts with Decisions' queue in the Author Area.

As you will see, the referees express considerable interest in your work, but have some significant criticisms and recommend a substantial revision of your manuscript before we can consider publication. If you are able to revise the manuscript along the lines suggested, which may involve further experiments, I will be happy receive a revised version of the manuscript. Your revised paper will be re-reviewed by one or more of the original referees, and acceptance of your manuscript will depend on your addressing satisfactorily the reviewers' major concerns. Please also note that Development will normally permit only one round of major revision.

We are aware that you may currently be unable to access the lab to undertake experimental revisions. If it would be helpful, we encourage you to contact us to discuss your revision in greater detail. Please send us a point-by-point response indicating where you are able to address concerns raised (either experimentally or by changes to the text) and where you will not be able to do so within the normal timeframe of a revision. We will then provide further guidance. Please also note that we are happy to extend revision timeframes as necessary.

Please attend to all of the reviewers' comments and ensure that you clearly highlight all changes made in the revised manuscript. Please avoid using 'Tracked changes' in Word files as these are lost in PDF conversion. I should be grateful if you would also provide a point-by-point response detailing how you have dealt with the points raised by the reviewers in the 'Response to Reviewers' box. If you do not agree with any of their criticisms or suggestions please explain clearly why this is so.

© 2022. Published by The Company of Biologists under the terms of the Creative Commons Attribution License (https://creativecommons.org/licenses/by/4.0/).

Reviewer 1

Advance summary and potential significance to field

The authors have previously shown that mixing embryonic stem cells (ESCs) and trophoblast stem cells (TSCs) can result in blastocyst-like structures ("blastoids") that also give rise to some primitive endoderm (PrE) cells. However, these structures previously showed little expansion of the PrE. Here the authors perform a screen to identify culture conditions to enhance the fraction of PrE-like cells in vitro, which may then support more efficient and further development of blastoid structures. They identify a PrE inductive medium and show that this supports further development of the blasted structures to a post-implantation-like morphology. This is an interesting system, which is well suited to such experiments, permitting the investigation of a large number of conditions. The authors study the effect of a variety of signalling molecules and inhibitors at different concentrations on PDGFRa expression. While this is a useful study, the authors describe the system as a model of the early mouse embryo but do not attempt to address or discuss the relevance of their findings in the context of the embryo. Thus, it is currently unclear how accurately this system and their findings recapitulate developmental events. I believe that the authors could strengthen this with some additional data and further discussion.

Comments for the author

ISSUES TO ADDRESS:
- There is no characterization of what happens in these structures over time and therefore it is unclear how this relates to specification of Epi and PrE in the in vivo blastocyst i.e. do PrE-like cells initially arise in aggregates in a salt and pepper manner before sorting to the outer edge of the aggregates? Are epiblast and PrE markers initially coexpressed in these conditions as in the embryo? Do PrE cells recapitulate the molecular expression pattern in the embryo?
- The authors identify a cocktail of signaling factors that appear to promote a PrE-like identity in vitro.
However, they do not determine whether these signals affect PrE specification in vivo. Currently, FGF is the signaling factor most clearly associated with Epi/PrE specification in the pre-implantation embryo. Addition of FGF to pre-implantation embryo culture promotes PrE specification and vice versa when FGF signaling is inhibited. The authors should attempt to validate the importance of these signals on PrE specification in the embryo by performing similar experiments, culturing early embryos in their PrE culture medium and its individual components.
- Here the authors show the importance of RA signalling in PrE-like induction in their system. However, it has been suggested that the onset of RA signalling in the embryo is during gastrulation (PMID: 22318625). Is there any evidence of earlier signaling or expression of appropriate components of this pathway at earlier times (e.g. from published single cell sequencing data of early embryos?).
- Similarly, the data shows that Wnt promotes PDGFRa expression and, surprisingly, has a stronger effect than FGF. However, Wnt signaling is not required for pre-implantation development (PMID:21554866). As Wnt induces mesoderm, could it be that in Fig. 1E, the effect of Wnt on PDGFRa expression is related to mesoderm differentiation?
- The authors conclude: "Our data support the idea that these two cell types are autonomously supporting the transition into the post-implantation stage of the Epi." – At the moment this statement seems to rely on morphology alone. This should be corroborated with some lineage markers e.g. downregulation of naïve pluripotency markers (e.g. Klf4) and upregulation of formative/primed markers (e.g. Oct6).
- P7: "We concluded that the Fgf and Wnt pathways regulate both the specification and the expansion of Pdgfrα + cells." How are the effect on expansion vs. specification of these molecules in the screen distinguished? The size of EBs treated with FGF or CHIR is not altered, which might suggest that there is not an effect on expansion? If claiming effects on expansion, the authors should look directly at proliferation under these treatment conditions e.g. by quantification of proliferation markers such as phospho histone H3.
- PDGFRa is not a PrE-specific marker but is also expressed within mesoderm cells. While the authors characterize the PDGFRa+ cells in the identified PrE-induction medium later in the manuscript, without further markers (such as Sox17-endoderm and Brachyury-mesoderm) in the

© 2022. Published by The Company of Biologists under the terms of the Creative Commons Attribution License (https://creativecommons.org/licenses/by/4.0/).

earlier treatments (Fig. 1E-J), it is not clear whether the observed effects are on PrE or mesoderm differentiation. This caveat should be acknowledged or further investigated.

- While there are clear benefits to the imaging analysis performed in the paper (to study the effect of these treatments on a per aggregate basis), some of the measurements are difficult to interpret. While 'yield' and 'number of PDGFRa clones' together does go some way to understand the effect on PDGFRa expression without any information about the size of the clones, the meaning of the data is still somewhat unclear.

Therefore, it would be valuable for the authors confirm some of the key results by bulk flow cytometry which will show exact cell numbers and percentages for each population.

MINOR QUERIES:

- Authors should be sure to add n values representing the number of independent experiments in the figure legends for each panel – it is currently missing in some places.
- The authors use 0.05 mM B-mercaptoethanol for their culture medium, which seems to be half the typical dose for ESC cultures. Is there a reason for this and, if so, they should state it in the methods?
- Correct/clarify meaning of P4: "enhanced the permittivity for PrE formation"
- Fig. 1D – Graph refers to number of GFP+ clones while legend refers to number of GFP+ cells. Should change to the correct one.
- P9: "Epi-specific Fgf4 and PrE-specific Fgfr2 were mutually expressed" – should this be mutually exclusively expressed? If so, perhaps could plot these gene levels from the same cells against one another to show this more clearly.
- Not always clear what the images represent e.g. Fig. 4C – is this a maximum intensity confocal projection?
- Fig. 5A: Not clear what is meant by 'survival' and how this is measured. Should be described in text and/or figure legend.
- Fig. 5B: more detail needed in figure legend. Does each point represent an individual cell aggregate?
- P16: "The molecular induction of the ESCs at the onset of blastoid formation did not affect the survival of Epi cells (96 h, 98% vs 100%, Figure 5A) but enhanced the potential of the PrE-like cells to expand (96 h, Gata6+, 53% vs. 10%, Figure 5A), an effect that depended on the initial number of PrE cells present in blastoids (Figure 5B)." – Not clear from this analysis that survival depends on number of cells, although it may be correlated. This should be reworded. Also, the authors should plot the number of cells at the beginning vs. at the end of the experiment, rather than number of cells at the beginning vs.
yes/no. This would give much more information on expansion/survival that currently cannot be gleaned.
- Fig. 5C: not clear what the alternative phenotypes are that don't fall into this category – 2D structures?
3D structures with only 1 cell type? Should include this in the graph or legend.
- Fig 5D: the authors should supply some higher resolution images/sections of these aggregates and show separate channels for IF as it is currently difficult to discern the structure from the data provided i.e. is there another cell layer inside the Oct4+ layer in lower left panel? F-ACTIN in upper right panel hinders clearly understanding the structure of this aggregate (hence also showing separate channels would help here).

Reviewer 2

Advance summary and potential significance to field

A new media to encourage primitive endoderm from small EB's of mESC.

Comments for the author

This is a reasonable contribution to a rapidly moving area, and should be publish with substantially more data analysis to compare with prior work, and ideally a few more experiments.

The authors develop a media to favor the development of Prim Endoderm (PrE) cells in small embroid bodies made from naive mESC. They show these structures place the PrE on the outside and the epiblast inside cavitates.

© 2022. Published by The Company of Biologists under the terms of the Creative Commons Attribution License (https://creativecommons.org/licenses/by/4.0/).

The authors in Fig 2 propose markers to define the PrE population and suggest further polarization to a VE like and Parietal endoderm-like cells. There is much more recent data that the authors do not cite which are important to situate their cells relative to the blastoderm and other claimants to the PrE state. It is also rather uncertain to what temporal stage of development their cell correspond. Specifically…
1. There is scRNA-seq data from the Hadjantonakis lab http://endoderm-explorer-app.us-east-1.elasticbeanstalk.com/ that should be compared with. They have many more cells that the earlier paper from Niaken's lab in 2015. Since the authors are using Seurat to analysis their sc-RNA-seq data they can easily integrate other such data sets and see how the combined data sets cluster as in Fig 2C-E. This would be much more convincing than picking certain genes.
2. There is a 2017 NCB paper from the Brickman lab, what purports to derive PrE cells from naive mESC, with a different recipe (+- insulin) and not in EB's. How do the authors cells compare? Of course it would be very interesting if signals between the future epiblast and PrE were essential to the conversion. Is there any evidence?
3. There are two recent papers, from the Zernicka-Goetz lab and Belmonte labs in Dev Cell and Cell respectively that generate blastocysts from extended pluripotency mouse ESC (the former added additional TSC and the Belmonte group did not) which are not mentioned. (Generation of Blastocyst-like Structures from Mouse Embryonic and Adult Cell Cultures, Self-Organization of Mouse Stem Cells into an Extended Potential Blastoid.).
Although these papers 'scoop' this submission, I definitely feel multiple approaches to the same nominal endpoint are useful to have in the literature. Since none of these structures develop further when implanted, more needs to be learned, so parallel work should be encouraged. That said prior work can not be ignored, and these papers too should be integrated with the gene clustering shown in Fig 2.
4. The cell sorting that places the PrE cells on the surface of the EB's, Fig 3, has been seen by others in EB's in a less organized way. But there are still many open questions. In the blastocyst, prior work of Saiz 2016 that is cited as well as bioRxiv 2019.12.27.889006; doi: https://doi.org/10.1101/2019.12.27.889006 e (that needs to be cited), shows that the transition from ICM to separate epiblast and PrE compartments takes place via cells that are double positive for Gata6 and Nanog (at a point when the blastocyst has a defined number of cells). Is this observed in the authors system? In Fig 4 there is data on Gata6 and Nanog separately but not the overlap as best I can tell, and only end point data and not as a function of time. Such data would give us both a time point and confidence that the path to the Epi and PrE state resembles that in vivo. The concern of Fig 4 is that their new protocol gives more PrE than doing nothing, a very low bar to pass since they selected factors with this in mind. Comparison with a real blastocyst is more interesting.
5. Alternatively one could use drugs or morphogens that have an effect on the PrE-Epi transition in the blastocyst and see if they have similar effects here. One such test was with Lif to show it impedes or blocks polarization and cavitation of the epiblast as in vivo.
Another more relevant test would be additional FGF4 or FGF/ERK inhibition. Does that govern the PrE/EPI ratio? The result is interesting in either case. If there is no effect, then the structures are plausibly post E4.5 when the corresponding cells in the blastocyst are committed to their fates. This is not a complicated experiment with some embryos on the side as controls. I think it should be done, given the large literature on this pathway in vivo.
6. The other interesting problem for which the authors could extract data, is growth coordination between epiblast and PrE. They do not have the optimal markers for this, but could they plot the areal density of the PDGFRa cells as a function of cyst radius for different times and all radii.

There are multiple minor problems in the writing:
1. The title or abstract must mention mouse somewhere. One can guess, but mammalian does not default to mouse.
2. In various places the authors list papers that show some effect of Lif, TGFb (both branches), Wnt, RA etc on the PrE/Epi ratio, to motivate their choice of screening factors.
More helpful would be one extended discussion with more detail. For instance which factors have a phenotype in the embryo following mutation. Which have an effect when applied to a blastocyst in vitro? And what is the effect, assayed how?
3. In Fig 4, I have no idea what if any tongues of the alluvial diagram Fig 4E are represented there or prior figures as actual cell images
In summary this paper should definitely appear, since no one has accomplished the ultimate goal of a live birth from a synthetic blastocyst (as has been done with synthetic oocytes in 2016), thus multiple protocols should be in the literature.

© 2022. Published by The Company of Biologists under the terms of the Creative Commons Attribution License (https://creativecommons.org/licenses/by/4.0/).

Reviewer 3

Advance summary and potential significance to field

In this manuscript Vrij et al., explore the potential of aggregates of mouse ES cells to generate Primitive Endoderm (Pr.End). The differentiation conditions they focus in, which they term as PrE induction conditions, allow the formation of embryoid bodies (EBs) containing cells of both epiblast (epi) and PrE lineages and are then applied to the blastoid system that has been developed in the Rivron lab. The early version of the system has been shown not to be effective in the production of PrEnd.

These results seem promising as some of their conditiond produce both epiblast and PrE lineage cells, also show a spatial organization similar to how they are in E4.5 mouse blastocysts and can initiate lumen (pro-amniotic cavity like structure) formation later.
The cocktain that they find to induce PrEnd and, in particular, the potential involvement of GPCRs is novel and potentially useful.

Comments for the author

This study is a contribution to the emerging field of synthetic embryology but should be considered a technical study rather than a research manuscript. Before publication it would be helpful if the authors could consider the following considerations
1. The authors mostly have used percentage to show the efficiency of the system. Since presenting data in this manner can be very misleading, the authors should also provide the exact number (like number of well with aggregates, number of blastoids formed, number of blastoids with both PrE and Epi lineage cells, how many replicates for each experiment etc) for all experiments (especially for the experiments involving blastoids). For the same reason, it is hard to understand how many times the author had to repeat the experiment to get to the number for example the ones they have shown in Figure 4B.
2. Figure 1E-1J; Authors have used agonists and inhibitors of different signalling pathways to test their effect on the emergence of PrE. However, in some instances agonist and activators of a given signalling pathway do not necessarily shown the opposite effect on PrE development. Moreover, despite activation or inhibition of some signalling pathways therer appears to be an improvement of PrE induction. In the absence of a statistical analysis it is not clear whether these changes are significant.
3. The authors have clearly shown that the PrE like cells in their system represent the post-implantation extraembryonic endoderm (XEn). However, the state of the epiblast like cells appears to be related to the pre-implantation epiblast (equivalent to E4.5 embryos). This creates a developmental gap between the two cell types within single aggregate, in contrast with the situation in vivo, when the differentiation of PrE into Visceral/Parietal endoderm and rosette formation in epiblast takes place simultaneously between E4.5 and E5.5. and rosette formation is influenced by PrE. Therefore, one wonders how this developmental gap between the two cell types is affecting the rosette development and blastoids formation in their system.
4. From the start, the authors argue in support of PrE-like cells generation in chemically defined serum free condition. Yet, they use serum in their medium for blastoids generation. Therefore, it is not easy to understand the point of using serum free condition during PrE induction when they ultimately end up using serum. Did the authors try to generate bastoids in serum free condition as well? Also, considering that PrE induction efficiency in serum (>90% EBs expressed PrE markers just with retinoic acid and LIF) was better than in N2B27 medium, why did they not perform all experiments on serum based medium rather than to switch between two media conditions.
5. Figure 5: Given such a low number of 3D structure formation, of which only 3 have lumen during in vitro blastoid culture, the data are not strong enough to claim that the PrE induced blastoids support the morphological features of post implantation embryos including pro-amniotic cavity like structure development during in vitro culture.
6. Initial cell number is quite crucial in these kind of experiments. The authors seeded 7 ESCs for PrE/Epi embryoid bodies and blastoid generation and 14 ESCs for Xen/Epi rosettes generation. Was there any particular reason behind using different number of cells between these experiments?

© 2022. Published by The Company of Biologists under the terms of the Creative Commons Attribution License (https://creativecommons.org/licenses/by/4.0/).

Their efficiency during XEn/Epi rosette EBs development was 94%. However, only 31% of blastoids showed 3D structure meaning even lower efficiency for the ones with rosettes like structure during in vitro culture. Could this lower rate of rosette formation in blastoid in vitro culture be attributed to the lower number of cells initially seeded which could potentially impact the mechanical forces and ultimately affect the development of structure like lumen, rosettes etc.

7. Based on point 5 of comments, I suggest to change the title of the manuscript as expansion is an overstatement of the modest results that they obtained

8. Naturally, the most crucial experiment that is missing is the test whether their PrEnd enhanced blastoids implant (something missing from the early version). This experiment is not needed if the manuscript is to be published as a technical advance/report but it would be good for the authors to comment on it.

First revision

Author response to reviewers' comments

Reviewer 1 Advance Summary and Potential Significance to Field:
This is an interesting study by the group of Nicolas Rivron, that contributes to the emerging field of synthetic embryogenesis. The manuscript presents a novel methodology that will be of interest for the field. Moreover, this methodology has the potential to improve existing blastoid models. I support the publication of this manuscript in Development.

We thank the Reviewers for addressing all these points and we believe this has led to a considerably improved manuscript.

Reviewer 1 Comments for the Author:
The following points need to be clarified:

1. Image analysis: the way this is presented throughout the manuscript is misleading. The graphs specify "count of GFP+ cells per EB", but the methods indicate that only a single Z plane within the EB was analysed. Therefore, the graphs show "count of GFP+ cells on a selected Z plane". This needs to be specified both in the graph and the figure legend. Panel 1A should also specify that even though the culture is in 3D the high-content analysis is in 2D.

We have added information in the figure legend that a 2D mid-focal plane is used for imaging and that a proxy for EB size is measured via the 2D projection area. We also changed this in Figure 1A.

2. Figure 1E-J: the conclusions of the authors do not match the data presented. They state that Nodal, Activin-A, Bmp4 or Tgf-b1 treatments reduced PE specification. However, none of these treatments had a significant effect. SB43 increased the number of clusters per EB but not the PrE yield, while A83, which also inhibits TGFbRI, had no effect. Altogether, their data seems to indicate that the TGFb pathway has no role in the formation of Pdgfra+ cells although the authors conclude otherwise.

The image data in this screen has been rigorously analyzed and the results that are depicted in this Figure adequately reflect the microscopy observations. Since the readout of this screen, as rightly addressed by the Reviewer previously, depended on the activation of a Pdgfra reporter we cannot make any conclusions from this initial screen regarding PrE specification beyond the observation of PDGFRa regulation. Moreover, the ANOVA test was run in an extremely strict fashion by including the three variables (PrE yield, clusters per EB and projection area) altogether for the compensation of variance between treatments. We did this because the three variables are not fully independent (e.g., a larger projection area may correlate to a higher number of Pdgfra+ cells by default). However, when a statistically significant result is obtained, such as for the number of Pdgfra+ clusters per EB in the Sb43 condition, it can be interpreted as important. Especially, since every data point here is already an average of ~400 EBs (one well with EBs).

However, we agree that the claim "Activin-A, Bmp4 or Tgf-b1 treatments reduced PE

© 2022. Published by The Company of Biologists under the terms of the Creative Commons Attribution License (https://creativecommons.org/licenses/by/4.0/).

specification" is too strong and may need to be rephrased. We adjusted the claim to "Activin-A and Tgf-β1 elicited a decline, albeit non-statistically significant, of either the yield or the number of Pdgfrα+ cells."

3. EPI-like identity of the structures: figure 2 focuses on the characterisation of the PE-like compartment in the structures, but the EPI-like compartment is not described. What is the pluripotent state of these cells? The data seems to indicate they have a mixed identity as they co-express Esrrb (naïve marker) and Otx2 (post-implantation factor). This is particularly relevant as the authors refer to naïve pluripotency exit throughout the manuscript, but it is not clear at present whether the process of naïve pluripotency exit happens prior to PE specification or after.

Indeed, the first scRNAseq data corresponds to structures in microwells 96 hours after seeding the cells and PrE/Epi induction. At this time point the Epi seems to have a mixed identity of naive and post-implantation epiblast. We have added GSEA compared to E5.5 mouse embryo epiblast which gives a similar negative enrichment score (-0.55) as when compared to E4.5 Epiblast (see below and Figure 2E). Thus the developmental window likely corresponds to the E4.5-E5.5 in natural embryos. This is also in line with the findings of an early mixed PE and VE identity for the PrE. In the second scRNAseq dataset we included 2i/Lif cells that show an initial naive pluripotent identity of the cells we use, thus the naive pluripotency exit does happen but it remains unclear exactly how this relates to timing in PrE specification. Likely, PrE specification occurs before the naive pluripotency exit, which is supported by findings shown in Figure 2A. Here, it can be seen that Gata6+ cells (PrE) can be found already 24 hours after induction.

Pdgfrα- population comparison to Embryo E5.5 Epiblast

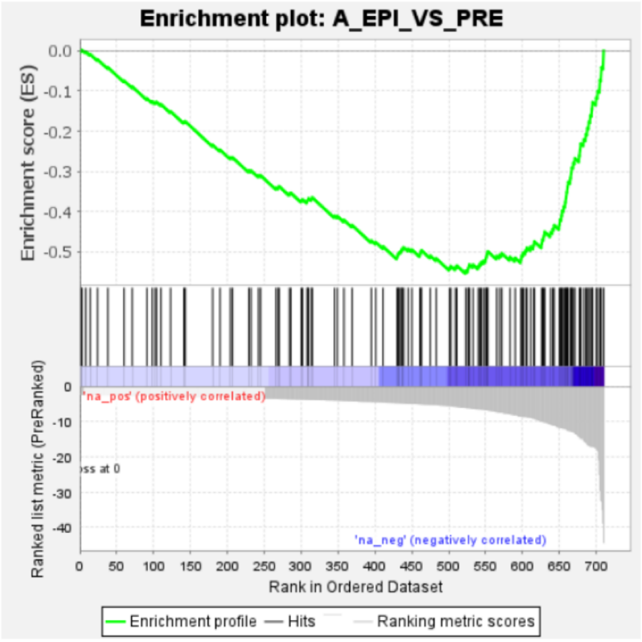

Dataset	Epi_E5.5
Phenotype	NoPhenotypeAvailable
Upregulated in class	na_neg
GeneSet	A_EPI_VS_PRE
Enrichment Score (ES)	-0.5531322
Normalized Enrichment Score (NES)	-1.9194896

We have included this data in Figure 2 of the manuscript and now mention that the Epi and PrE reflect the peri-implantation blastocyst-stage Epi and PrE.

4. Dynamics of PrE segregation: figure 2A shows an example at 24 hours, while figure 2B shows an example at 96 hours. A panel showing examples of structures every 24 hours (as shown in the quantifications of figure 1A) should be included.

The 24 hours images in Figure 2A are included as an example for the double positive (Gata6+ and Nanog+) cells that are found within structures. We added z-stack tiles for all colors and all time

© 2022. Published by The Company of Biologists under the terms of the Creative Commons Attribution License (https://creativecommons.org/licenses/by/4.0/).

points in supplementary Fig. S6.

5. Figure 2F: adding the comparison to PE would be informative. At the moment only the comparison to VE is shown.

This would indeed be very informative. However, we are unaware of any gene list for E5.5 PE. Likely, due to that PE tissue is very difficult to isolate from embryos within the first 48 hours after implantation.
However, for comparative purposes we did compare our PrE and PE and VE-like subpopulation data to E6.5 embryo data (Blanca Pijuan-Sal et al., 2019) using GSEA. Since we could insert only the log2FC values and the ranked gene set (thus no p-values) these results cannot be directly compared to the more reliable GSEA results in Figure 2F. Overall, the PrE cluster (Pdgfrα+ cells) and the VE-like and PE-like subclusters appear enriched in genes of the E6.5 PE, of which the PE subcluster contributes the most. These results support the findings that the Pdgfrα+ cells have an extraembryonic endoderm identity primed for both PE and VE.

Compared to E6.5 PE (vs E6.5 Epi, (Blanca Pijuan-Sal et al., 2019)

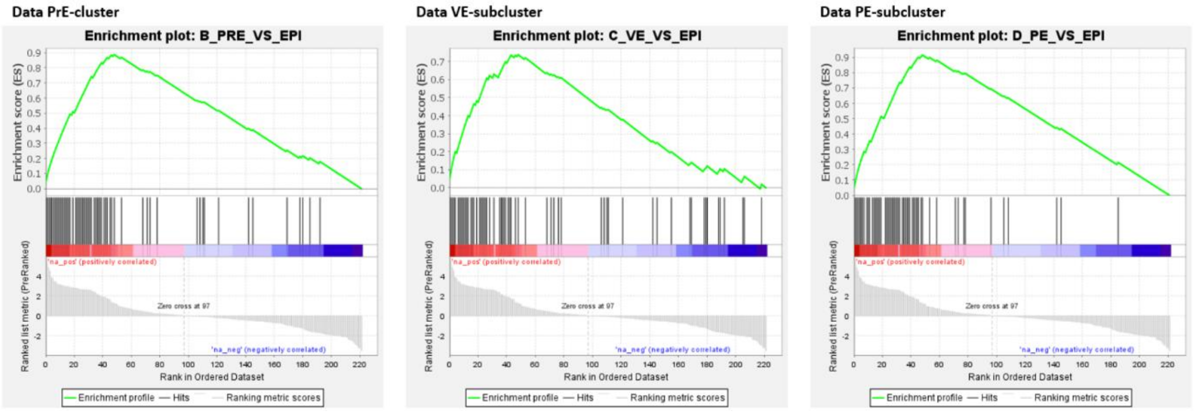

6. The statement “within 24 hours after induction, double-positive cells and a few double negative cells emerged” is misleading. Double positive cells are as few as double negative cells (approximately 10% in both cases).

We have adjusted the sentence to “within 24 hours after induction, double-positive cells and double negative cells emerged”.

7. The authors conclude that the Tgfb pathway is required for the initiation of VE specification, but the data presented to support this claim is really minimal (only expression of Dab2 and Runx1). The authors should either tone down their claims, or perform additional experiments.

We have toned down thes concluding statement to “a possible implication of the Tgf-β pathways in the initiation of VE.”

8. The presence of VE-primed and PE-primed cells within the structures is very interesting. Further information would be gained by performing immunofluorescence staining for specific markers. Do PE-primed cells lose contact with the epiblast?

We agree with the Reviewer that this is very interesting. We believe that the use of XEn/Epi EpiC in studying PE/VE specification dynamics would require much more analysis and we would prefer to do this carefully in a follow-up study. Importantly, we are already more than 2000 words over the maximum word count in our current manuscript.

9. XEN/EPI rosettes: the name needs to be changed. A rosette refers to the organisation of epithelial cells in a polarised conformation towards a central point, with their apical domains constricted. This stage precedes the formation of a central lumen, when the apical domain expands, and the cells lose their characteristic wedge shape. What the authors show are examples

© 2022. Published by The Company of Biologists under the terms of the Creative Commons Attribution License (https://creativecommons.org/licenses/by/4.0/).

of lumens, not rosettes.

We agree with the Reviewer that the majority of structures depicted in the manuscript correspond to the Extraembryonic Endoderm/Epiblast epithelialized pro-amniotic-like cavity (XEn/Epi EpiC) stage. We have adjusted the naming accordingly throughout the manuscript to XEn/Epi EpiC, while only using the term Rosette to describe the Epi with a polarized conformation but before pro-amniotic cavity formation.

10. Figure 3F: this shows that in some structures there are multiple lumens. This is an interesting finding that is not commented in the results section. Is there a correlation between size and the multi-lumen phenotype? This would be along with the findings of Orietti et al, Stem Cell Reports, 2021.

This is an interesting comment. We checked our data again but since multi-lumen structures occur at very low frequency we can not reliably correlate it with the total size of structures using our current data sets.

11. Figure 3H: the authors mention that under LIF culture Podxl did not localise to the apical membrane in the VE-like compartment. This is not the case. In the image shown in Figure 3H Podxl clearly localises apically in VE-like cells.

We agree with the Reviewer. In the “No Lif” condition Podxl is found apically but also bilaterally on the majority of cells. Thus, we rephrased the sentence to “the absence of Podxl within the Epi-like cells and the arched bilateral/apical location of Podxl in the XEn-like cells.”

12. Figure 3I: as a control, the authors should show the Nodal KO structures before the switch to N2B27. Moreover, in the images shown it seems that the VE- like layer is also multi-layered in the control. Is this the case? It is difficult to conclude without a DAPI staining? Could the authors quantify the incidence of a multi-layered VE in the different experimental conditions?

We have performed antibody staining for PrE (Pdgfra) and Epi (Nanog) PrE-induced structures formed with both the Nodal KO -/- line and its corresponding WT control. At 72 hours after induction we observed no apparent difference in morphology of PrE nor Epi. This data is included in the supplementary information (Fig. S15).

© 2022. Published by The Company of Biologists under the terms of the Creative Commons Attribution License (https://creativecommons.org/licenses/by/4.0/).

PrE-induced EBs, WT control, 72 hours

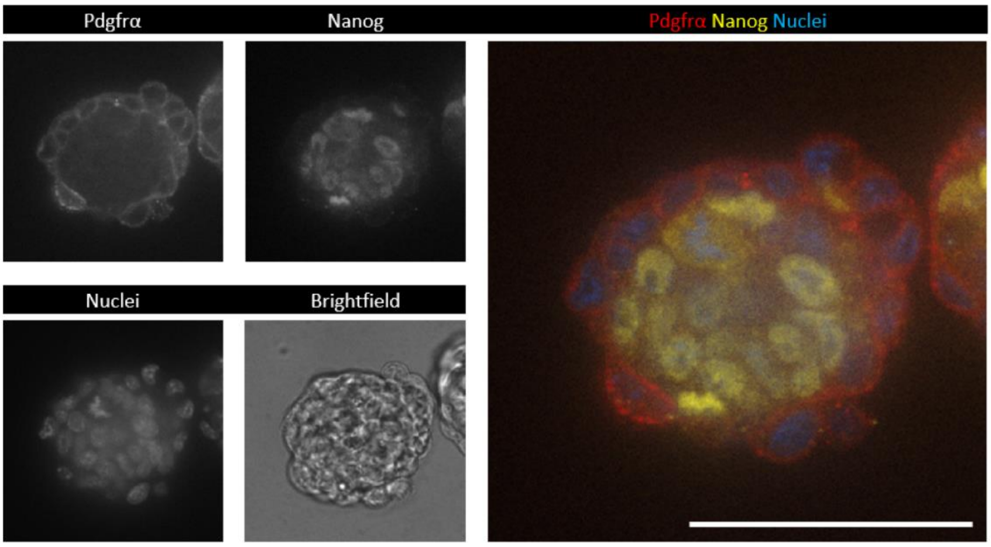

PrE-induced EBs, Nodal KO -/-, 72 hours

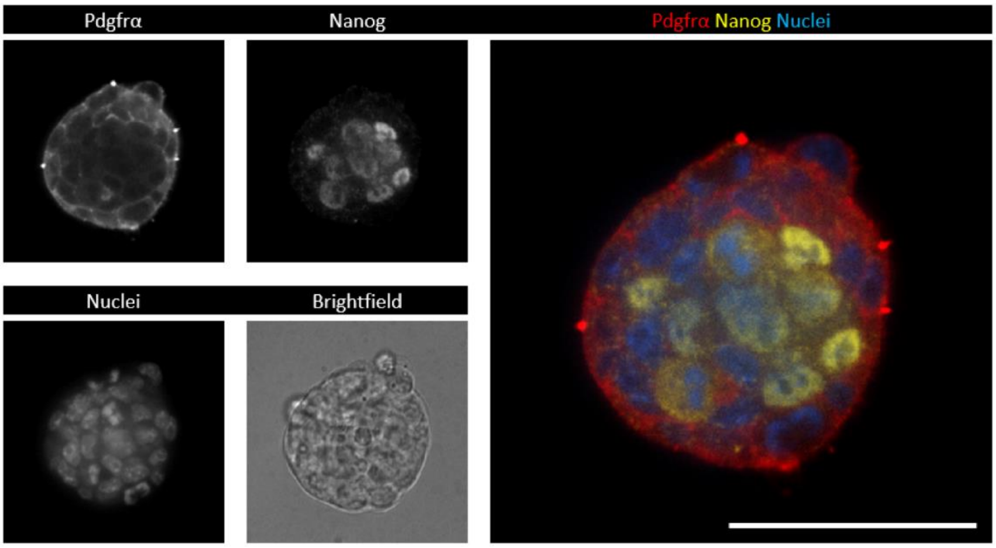

We also counted the number of single and multi-layered XEn layers and whether the XEn layer was (partly) delaminated from the Epi (total of 32 structures each) in the V6.5 ESC line with double KO for Nodal (-/-) and its corresponding WT control (+/+). This data is included in Figure 3 (3L).

© 2022. Published by The Company of Biologists under the terms of the Creative Commons Attribution License (https://creativecommons.org/licenses/by/4.0/).

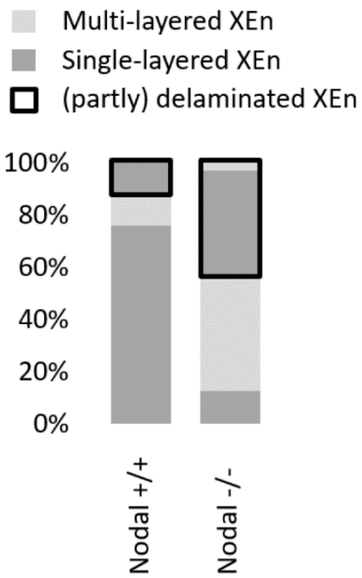

13. The appearance of mesoderm-like cells upon culture in N2B27 is very interesting, but could be characterised with a bit more detail. At the moment the analysis presented is solely based on sequencing data. Do anterior-like and posterior-like fates emerge within the same structure? Are there cases in which the VE-like cells do not surround the entire EPI-like compartment, and could this maybe explain the emergence of mesoderm-like cells?

We performed antibody staining for Brachyury and observed that 14% of XEn/Epi EpiCs (+64 hours) contained Brachyury-positive inner cells originating from an epithelium-like tissue. Additionally, we performed antibody staining on the minority (20%) of PrE/Epi-induced structures that did not form an epithelialized Epi with pro-amniotic-like cavity (EpiC) but remained instead with an amorphous cell clump engulfed by a XEn-like cell layer. Of note, these structures were not included for single cell RNA sequencing analysis.
We observed that these amorphous cell clumps were fully Brachyury-positive. Notably, a XEn-layer was present in all these structures and did not appear to be delaminated from the inner cells, which indicates that there is no correlation between the absence or a delaminated XEn-layer and posterior epiblast fate. This suggests that Epi-VE interactions are sufficient to initiate part of the gastrulation program. However, we cannot rule out that in a subset of structures (that do not cavitate) Chir may have primed cells towards mesoderm fate during initial induction, which as a result may prevent pro-amniotic cavity formation.

We have included the above mentioned data in Figure 4 and Figure S18.

© 2022. Published by The Company of Biologists under the terms of the Creative Commons Attribution License (https://creativecommons.org/licenses/by/4.0/).

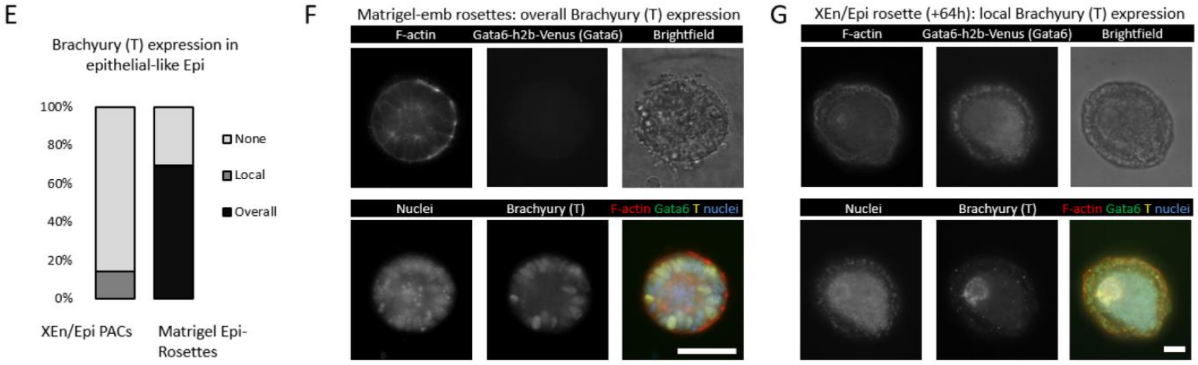

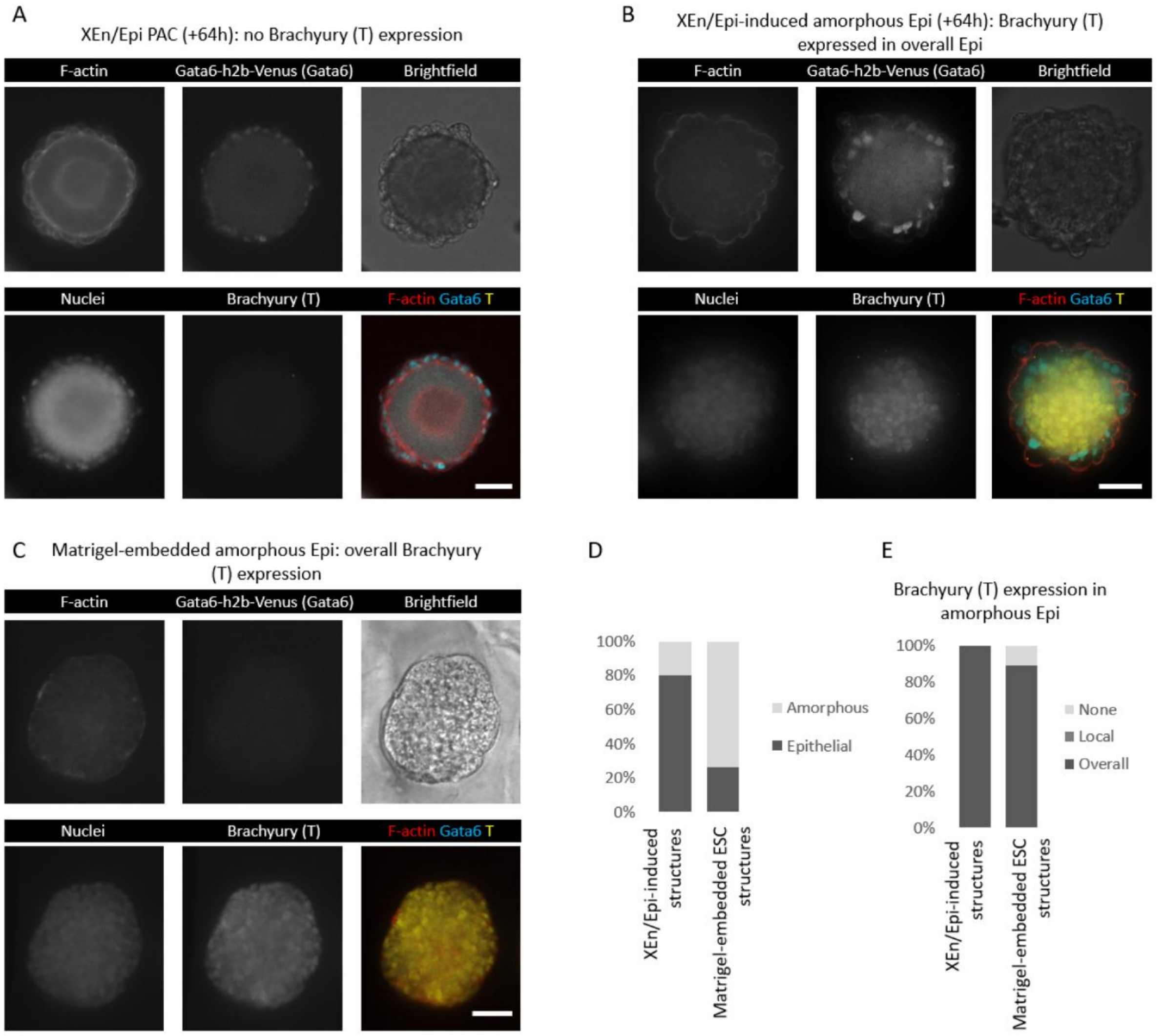

14. Figure 6D: this structure is completely disorganised. Oct4 positive cells are shown in what would be the ExE-like compartment (based on the brightfield image) and only in a subset of cells within the Epiblast-like compartment. What criteria have the authors used to classify that structure as organised? Could they provide more examples and stain for the TE compartment? Based on the images they present is not clear whether an ExE-like region exists.

We did not identify the formation of ExE-like regions, thus with “organized” we mean a central lumen surrounded by a (pseudostratified) Epi-like cell layer and XEn, thus pro-amniotic cavity-like but without the adjacent ExE. The majority of blastoids when grown out in IVC medium do not form such pro-amniotic cavity-like structures and either formed a 2D cell layer (no embryonic

© 2022. Published by The Company of Biologists under the terms of the Creative Commons Attribution License (https://creativecommons.org/licenses/by/4.0/).

structure) or a 3D structure (3D non-organized) that did not progress to structures with a pro-amniotic-like cavity. Somehow the TE does not progress, which can either be related to suboptimal post-implantation outgrowth culture conditions (e.g., handling, fluorescence microscopy to pick the blastoids with Gata6+/Pdgfra+ cells, IVC medium) or sub-potent TE-like tissue in our blastoids.

To emphasize the absence of the ExE we added the following phrase in the manuscript "..however, ExE-like tissue formation appeared absent."

15. In the discussion the authors state "one attractive possibility raised by this study is that the Epi proliferation and morphogenesis to form the amniotic cavity cannot occur unless the PrE deposits the required basal lamina". This is indeed the case as shown in laminin KO embryos that do not develop post-implantation and present an aberrant peri-implantation morphogenesis (Miner et al, Development, 2004 and Smyth et al, J Cell Biol, 1999).

We have added this information including the references in the discussion.

Minor comments:

1. Conclusion of figure 1: inhibition of GSK3b/bcatenin facilitates the generation of Pdgfra+ cells. This statement is confusing, as GSK3 inhibition leads to bcatenin activation.

We have adjusted this to .."inhibition of GSK3β and the Tgfβ pathway facilitate.."

2. What is the rationale to study GPCR ligands? Could they have a functional role in the embryo? The section on GPCR ligand screening comes out of the blue.

The rationale was instigated by a previous study that found cAMP modulates Pdgfrα expression in EBs. We have this information in the manuscript.

3. Figure 2F: there is a typo. The second plot should say "comparison to Embryo E5.5 VE".

We have changed this accordingly

4. Why do the authors isolate Pdgfra+ cells by FACS using an antibody when they have the reporter line? Why sometimes do they use a Gata6 reporter instead of the Pdgfra reporter?

We have seen using immunofluorescence that the Pdgfra reporter is not always switched on in cells that have Pdgfra present in their membrane. We found later that the Gata6 reporter line is more reliable.

5. Last section results: when referring to Oct6 expression, the figure is 6F, not 6E.

We have changed this accordingly

Reviewer 2 Advance Summary and Potential Significance to Field:
The authors make use of their previously characterised model system, blastoids, in which blastocyst-like structures can be generated from trophoblast stem cells and embryonic stem cell lineages. Building on this model, they probe the mechanism by which the Epiblast and extra embryonic endoderm develop. The authors are able to uncover how reciprocal interactions between the epiblast and primitive endoderm are important to permit the development and morphogenesis of the early post-implantation embryo.

This paper is an exceptionally useful addition to the field, and highly significant. In one facet, it shows how in vitro approaches (which are complementary to in vitro studies) can dissect the mechanisms for cell lineage allocation in a highly controlled and scaleable system. The authors' use of microcells show how reproducibility between blastoids was foremost on their minds, and does well to address the concerns some people in the field have regarding reproducibility in the 'organoid' field.

© 2022. Published by The Company of Biologists under the terms of the Creative Commons Attribution License (https://creativecommons.org/licenses/by/4.0/).

Reviewer 2 Comments for the Author:

I believe the authors have done a very good job of addressing the previous reviewers' comments (going by their rebuttal). I am very happy with the sections on data reproducibility. The majority of the figures are clear, and the conclusions in my mind are backed up well by the highly quantitative data. Regarding Fig. 6, the top portion feels slightly chaotic; I wonder if the table could be placed elsewhere, or formatted graphically? It is an important table to show though, and I'm happy the authors have provided these data; it slightly messes
with the flow of the figure.

We reformatted the table in order to make it less chaotic and improved the flow of the figure.

Minor points follow...

Materials and Methods
1 - Microscopy: Would the authors be able to add a bit more detail on the light-source and ex/em filters for their widefield?

We have added these details in the materials and methods section.

2 - Immunofluorescence: Although 'paraformaldehyde' is commonly used to describe the solution, it describes the solid; if I'm right, it ought to be '4% formaldehyde' solution.

We have changed this to ‘formaldehyde’.

3 Antibodies: I'd like to thank the authors for putting in all this information, especially on the dilutions. However, could the authors put this in table format? it's much easier to read this way.

We have placed this information in a table format.

I am also happy to see that the ss transcriptomics are available in GEO, however regarding the other data availability, I'm not to enthused with the "data available on request". I understand if they aren't able to do the, but would it be possible for the authors to deposit as much data as they can (e.g. microscopy images, replicates etc) on databases such as OMERO or something similar?

Via the DataVerseNL platform we made available the raw image data for Figure 2G, which contains many Rosettes over time (24, 48 and 72 hours after flushing them out from the microwells). The temporary link to access the data is: https://dataverse.nl/privateurl.xhtml?token=04c07d68-ad09-468d-8cb7-c5d5a1d491c2 The final DOI will be: https://doi.org/10.34894/GSOHSD

Reviewer 3 Advance Summary and Potential Significance to Field:
The authors clarify conditions for maintaining mouse ESC, epiblast and primitive endoderm cells in coculture, and suggest some interactions between the cell types that are necessary for development of the blastocyst. Multiple labs are playing in the area, noone has made a blastocyst that develops in vivo, but its an important problem, and multiple approaches need to be published.

Reviewer 3 Comments for the Author:
The authors have done a good job of responding to my questions/comments in my first report. I do not find in the main text or SI, the figure in Item #5 of their response to referee 2, showing effects of added FGF and its inhibition to proportion of XEn. That should be put somewhere and mentioned in main text.

We have included this data as a supplementary figure (Fig. S9) and referred to it in the main text.

The paper should be published.

© 2022. Published by The Company of Biologists under the terms of the Creative Commons Attribution License (https://creativecommons.org/licenses/by/4.0/).

Second decision letter

MS ID#: DEVELOP/2020/192310

MS TITLE: Pendulum of inductions between the epiblast and extraembryonic endoderm supports post-implantation progression.

AUTHORS: Erik Vrij, Yvonne Sholte Op Reimer, Javier Frias Aldeguer, Isabel Misteli Guerreiro, Bon-Kyoung Koo, Clemens van Blitterswijk, Jop Kind, and Nicolas Rivron
ARTICLE TYPE: Research Article

I am happy to tell you that your manuscript has been accepted for publication in Development, pending our standard ethics checks.

Reviewer 2

Advance summary and potential significance to field

see my prior report

Comments for the author

all OK publish

© 2022. Published by The Company of Biologists under the terms of the Creative Commons Attribution License (https://creativecommons.org/licenses/by/4.0/).
